# Breeding progress, genotypic and environmental variation and correlation of quality traits in malting barley in German official variety trials between 1983 and 2015

**DOI:** 10.1007/s00122-017-2967-4

**Published:** 2017-08-18

**Authors:** Friedrich Laidig, Hans-Peter Piepho, Dirk Rentel, Thomas Drobek, Uwe Meyer

**Affiliations:** 1 Bundessortenamt, Osterfelddamm 80, 30627 Hannover, Germany; 2 Biostatistics Unit, Institute of Crop Science, University of Hohenheim, Fruwirthstrasse 23, 70599 Stuttgart, Germany

## Abstract

**Key message:**

**Evaluation of breeding progress for spring barley varieties in Germany showed that both grain yield and malting quality were considerably improved during the last 33** **years, and that genetic effects of protein concentration and malting traits were not associated.**

**Abstract:**

Based on historical data, this study aimed to investigate yield potential and malting quality of 187 varieties tested and released in German registration trials to evaluate the value for cultivation and use (VCU) during 1983–2015, and to quantify the environmental variability and the association among traits. We used mixed linear models with multiple linear regression terms to dissect genetic and non-genetic trend components. Grain yield increased by 43% (23.4 dt ha^−1^) in VCU trials and 35% (14.0 dt ha^−1^) on-farm relative to 1983. All yield components contributed significantly. Malting quality was also considerably improved by 2.3% for extract content up to 25.1% for friability, relative to 1983, nearly completely due to new varieties. Total variability of individual traits was very different between traits (2.4–24.4% relative to 1983). The relative influence of genotypes on total variation was low for grain yield and its components, whereas it was considerably larger for other traits. We found remarkable differences between phenotypic and genetic correlation coefficients for grain yield and protein concentration with malting traits. The observed positive phenotypic relation between grain yield and malting quality can be attributed to a shift of selection and environmental effects, but genetic correlations showed a negative association. Genetic effects of protein concentration and malting quality were not correlated indicating that both were not genetically linked. Considerable yield progress and improvement of malting quality were achieved despite of their weak to moderate negative genetic dependence.

**Electronic supplementary material:**

The online version of this article (doi:10.1007/s00122-017-2967-4) contains supplementary material, which is available to authorized users.

## Introduction

**Fig. 1:**
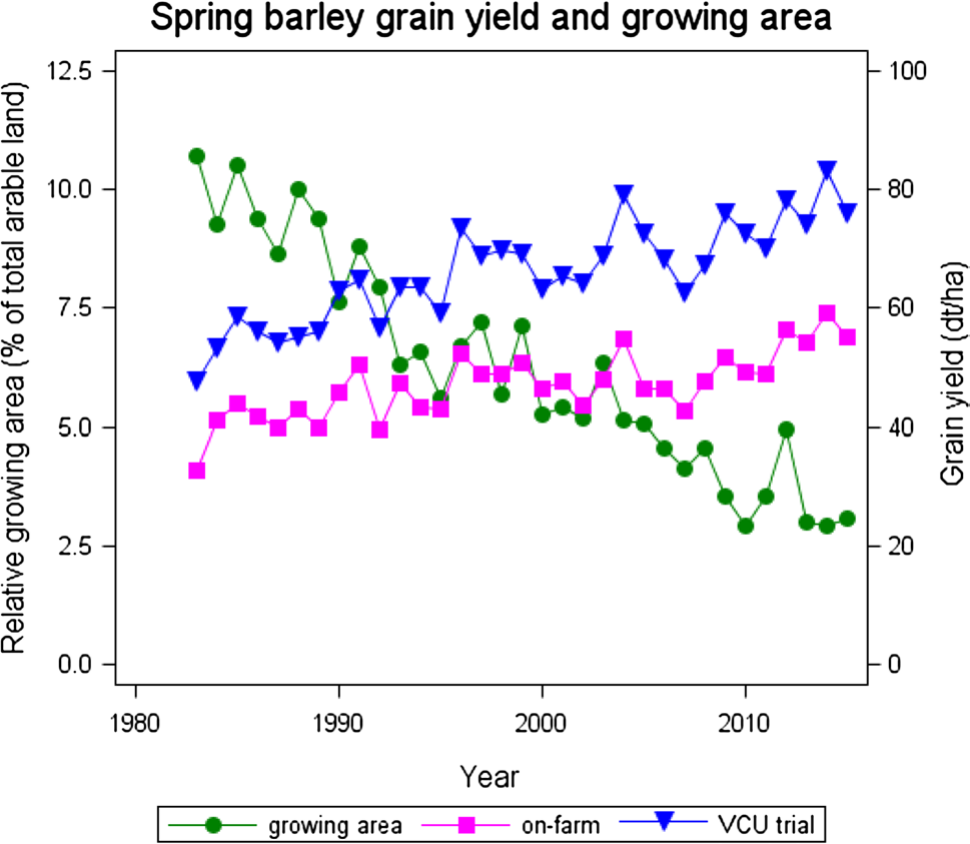
Spring barley growing area, trend in VCU trails and on-farm 1983–2015. *YEAR* calendar year, *On*-*farm* national on-farm average yield, *VCU*-*trial* adjusted overall year means [effect Y_*j*_ in Eq. (4)]

Spring barley is a very important field crop for the brewing industry in Germany. In 2015, the national grain production was 1947 thousand tons. During the last 3 years, approximately 67% of harvested grain was delivered as malting barley (Braugersten-Gemeinschaft 2016a).The growing area in Germany declined drastically from about 11% in 1983 to nearly 3% of total arable land in 2015 (Fig. 1), which corresponds to about 371,000 ha (StatJ 2015). General quality requirements for malting barley are a protein concentration in grain of at most 11.5%, and a grain fraction with kernel size >2.5 mm of at least 90% (Bundessortenamt 2016). Further quality specifications may be required depending on the processor. If these requirements are not met, grain lots are usually classified as of fodder quality which fetches a lower market price. The average market price for spring barley with brewing quality in 2011–2015 was 19.90 € per dt and with fodder quality 16.50 € per dt (Erntebericht 2015).

The use of ‘Hana-type’ barley varieties as donor, imported from Moravia in the early 1900s, increased malting quality in Germany considerably (Lekes 1990; Lekes 1998; Grausgruber et al. 2002; Fischbeck et al. 2008). A further major step towards higher yield and quality was achieved by the introduction of semi-dwarf varieties based on the variety ‘Trumpf’ in early 1970s. Due to this type of variety plant height was reduced by about 10 cm (Lekes 1990; Baumer et al. 2000; Fischbeck et al. 2008). Shorter varieties had the advantage that they were less prone to lodging, and allowed growers to better control nitrogen supply to obtain optimal nitrogen concentration in grain, which is very important for an optimal brewing process, and hence for a good beer quality (e.g., Varvel and Severson 1987; Arends et al. 1995; Eagles et al. 1995; Herz 2011). Lekes (1998) reported that during the last 35–40 years the most significant breeding progress was achieved in the reduction of plant height, as well as the increase of ear density and harvest index. A new phase of breeding for high malting quality began with the introduction of the micro-malting method in the 1960s, which allowed a directed selection for processing quality based on small samples (Baumer et al. 2000). Before this technique was available, early-generation breeding was based mainly on the so-called hand grading (‘Handbonitur’), and later additionally on the more objective assessment of the traits grain fraction with kernels >2.5 mm, protein concentration and extract content.

The progress achieved in grain yield for European spring barley was reported in numerous studies (Peltonen-Sainio et al. 2009; Psota et al. 2009; Baumer et al. 2000, 2004; Lillemo et al. 2010; Grausgruber et al. 2002; Mackay et al. 2011; Oberforster and Werteker 2011; Rijk et al. 2013). The reported annual increase in grain yield due to new varieties since 1950 was in the range between 0.28 and 0.79 dt ha^−1^. Most of the studies on malting quality are based on data from vintage trials, i.e., trials with older varieties replanted for only a few growing seasons (e.g., Rasmusson and Glass 1967; Rutger et al. 1967; Wych and Rasmusson 1983; Grausgruber et al. 2002; Ogushi et al. 2002; Nielsen and Munck 2003; Nielsen 2003; Passarella et al. 2002; Condon et al. 2009; Schmidt et al. 2016); only a few studies use data from historical trials (Baumer et al. 2000; Psota et al. 2009; Oberforster and Werteker 2011; Matthies et al. 2014).

Oberforster and Werteker (2011) found no unfavorable phenotypic inter-varietal relations between grain yield and essential quality traits, which seems of advantage for barley breeding towards improved malting quality. By way of comparison, in winter wheat a strong negative relation between grain yield and baking quality hampers improvement of baking quality (e.g., Laidig et al. 2017). For the most important malting trait, i.e., extract content, considerable increases per year were found of 0.054% by Passarella et al. (2002), of 0.0641% by Psota et al. (2009) and of 0.086% by Baumer et al. (2000). Extract content in Bavarian barley trials rose to 84.3% during 1991–2000 as reported by Baumer et al. (2000), but the authors conjectured that a further increase above the 84% level would be very unlikely.

Malting quality is of a complex nature and is described by a series of traits, which follow a complex mode of inheritance (Ogushi et al. 2002; Matthies et al. 2014; Schmidt et al. 2016). Variation of individual traits is influenced by genotype, the environment in which the variety is grown, and the malting and brewing process. For optimal planning of field trials and laboratory tests, it is beneficial to know the relative magnitude of individual components of variation, especially how much of it is attributable to genetic variation and how much to genotype × environment interaction (Kleinknecht et al. 2016). Molina-Cano et al. (1997) reported on 11 studies conducted so far on variation of malting quality, revealing contradictory results regarding the existence and magnitude of genotype × environment interaction. Generally, the environmental effects had more influence on total variation than their interaction with genotypes.

As malting quality is of a complex multidimensional nature, principal component analysis is a useful method to graphically display association between traits (e.g., Munck 1991; Molina-Cano et al. 1997; Rey et al. 2009; Nielsen and Munck 2003; Cozzolino et al. 2012). Principal component plots give an easy-to-interpret picture about the correlation structure of the components for malting quality. Many studies reported correlation coefficients between individual traits of grain and malting quality (e.g., Rutger et al. 1967; Arends et al. 1995). The results are quite different, however, depending on geographical region, selection of genotypes, seasons and crop management. Condon et al. (2009) reported from a study with 98 barley genotypes, including historical varieties, parent and elite lines varieties, and breeding lines, that significant correlations among traits were most likely due to simultaneous selection. Breeder’s knowledge of the association between visible traits used for selection in early breeding stages, e.g., seed size, and hidden traits assessed in a very late stage, like extract content, may be of help because of their effect due to indirect selection (Munck 1991).

The objective of our study is to evaluate the progress made in malting barley breeding based on varieties tested in official VCU trials in Germany between 1983 and 2015, estimate the influence of genotypic and environmental effects on variation of agronomic and quality traits and describe the phenotypic and genetic relationships between traits.

## Materials and methods

### Dataset

Data on grain yield, its components, and traits for grain and malting quality from German official spring barley variety trials were analyzed over the period from 1983 to 2015 (Table 1). In our data set, we used 187 released varieties, including 37 standard varieties. The statutory testing period for newly submitted varieties in registration trials is 3 years, whereas a standard variety stays in trials about 7 years on the average. The data available for individual traits may be different for several reasons. For example, quality samples from a trial had to be excluded if protein concentration was above malting quality level, extreme observations had to be removed from the data, or data were lacking due to technical problems during harvesting. The number of observations per trait was between 5266 for friability and 8594 for grain yield. The data comprised 33 years (1983–2015). Within 1 year three trial series were conducted. Series 1, 2 and 3 include varieties being in the first, second and third testing year, respectively. Data were collected from eight locations per trial series and year. The data set was very non-orthogonal with respect to variety–year combinations, whereas the variety–location combinations were orthogonal within year and trial series, i.e., all varieties were grown together at all locations within the same year and trial series. Only about 1.6% of the possible variety–location–year combinations are present. A detailed description of the dataset is provided in Electronic Appendix Tables S1 and S2.

**Table 1:** Investigated traits

Trait	Abbreviations	Unit	Test type	Description
Yield components				
Grain yield at 86% dry matter	GRAIN_Y	dt ha^−1^	Field trial	Assessed on treatment with nitrogen application at malting barley level. Trial layout as split-plot, main plots in complete blocks (2 treatments), varieties in subunits. Average harvested plot size about 10 m^−2^
Single ear density	EAR_D	ears m^−2^	Field trial	Calculated from a row of one meter length of one single plot. All varieties at the same location are sown at equal number of kernels per square meter according to local conditions in the range from 250 to 480 kernels per square meter
Number of kernels per ear	KERNLS_E	kernels ear^−1^	Field trial	Calculated from thousand grain mass and single ear density
Thousand grain mass at 86% dry matter	TGM	g (1000 kernels)^−1^	EBC -Method, MEBAK (2006) (1.3.2)	
Grain quality				
Grain fraction > 2.5 mm	GRAIN2.5	%	EBC-Method, MEBAK (2006) (1.3.1)	The grain fraction with kernel size >2.5 mm of the raw grain
Hectoliter weight (test weight)	HECTOL_W	kg hl^−1^	EBC-Method, MEBAK (2006) (1.3.3)	An important criterion for exterior grain quality
Crude grain protein concentration [% of dry matter]	PROTIN_C	%	EBC-Method, MEBAK (2006) (3.1.4.5.1.1)	High protein concentration reduces quality with respect to extract content and malt solution
Malting quality				
Extract content in dry matter (%)	EXTRCT_C	%	EBC-Method, MEBAK (2006) (3.1.4.2.2)	Extract content indicates the fraction of soluble substances (particularly starch and protein) in the wort, and it is considered to be the most important characteristic for brewing quality
Malting loss	MALTNG_L	%	Calculated	Malting loss is calculated as the difference between grain dry matter and dry-malt in dry matter as percent of total grain dry matter. It is composed of energy loss due to respiration during germination and material loss due to rubbed-off freshly germinated roots after drying
Friability	FRIABLTY	%	EBC-Method MEBAK (2006) (3.1.3.6.1)	Friability measures the fraction of malt crushed by a screen drum and it indirectly indicates the degree of cytolytic malt solution. Higher friability values mean a better cytolytic solution of the malt
Viscosity	VISCOSTY	mPas	EBC-Method, MEBAK (2006) (3.1.4.4)	High viscosity of the wort points to low cytolytic malt solution. It further gives evidence as to the expected lautering time and foam stability of beers. Good malting barley should show low viscosity values
Protein solution degree (Kolbach value)	PROTIN_S	%	EBC-Method, MEBAK (2006) (3.1.4.5.3)	Protein solution degree is the nitrogen in the wort as per cent of total nitrogen in malt. It represents the proportion of solved protein in the wort. Malting barley should have values in the upper range
Final attenuation degree	ATTENUTN	%	EBC-Method, MEBAK (2006) (3.1.4.10.1.2)	Final attenuation degree expresses the sum of all by brewer’s yeast fermentable material as per cent of extract content in the wort. A high final attenuation degree is desired

*EBC* European Brewery Convention, *MEBAK* Mitteleuropäische Brautechnische Analysenkommision

For laboratory tests, bulked samples from replications were taken from each trial series every year. Quality was assessed in three different laboratories, i.e., the Versuchs- und Lehranstalt für Brauerei in Berlin e.V. (VLB), the Research Center Weihenstephan for Brewing and Food Quality (RCW) and the Bayerische Landesanstalt für Landwirtschaft Freising (LfL). VLB assessed all grain quality traits. Analysis of malting traits was split between the three laboratories in such a way that each laboratory always analyzed the complete set of locations for a trial series within the same testing year. Traits for grain and malting quality were analyzed according to “Methodensammlung Mitteleuropäische Brautechnische Analysenkommission” (MEBAK 2006) (Table 1).

Beginning with harvest year 2002, the malting time was reduced for all trial series from 7 to 6 days, which may have affected extract content, malting loss, friability, viscosity, protein solution degree and final attenuation degree. This change allowed savings in time during the malting process due to varieties with higher diastatic power. In 2012, there was a further change in mashing process from stepwise increasing temperature (45° to 70 °C) to an isothermal 65 °C process beginning with varieties tested for their first year in 2012. Varieties with their first year in trial 2011 were still tested under the old regime ending 2013. As results from both mashing variants are not comparable, we only used data up to 2013 for extract content, protein solution degree, viscosity and final attenuation degree.

To avoid biased results, we checked data thoroughly for consistency in structure over time before carrying out analysis. Inconsistent data structures may have occurred due to changes in assessment of a characteristics’ scale of measurement or structure of trial series. To identify such inconsistencies, we manually screened historic testing reports and trial plans up to 1991. In later years, the necessary information was already stored with the trial data. The data were further checked for recording errors and outliers by calculating standardized residuals based on Model (1), (2) and (3) described in the next sub-section. Observations with standardized residuals greater than ±5.0 were excluded from further analysis. 125 (0.14%) of all observations exceeded the threshold and were therefore excluded.

### Statistical analysis

*Model for genetic and non-genetic trend* We used a standard four-way model with factors genotype, year, trial series and location:

1 $$ y_{ijkl} = \mu + G_{i} + Y_{j} + (YT)_{jk} + L_{l} + (YL)_{jl} + (YTL)_{jkl} + (GY)_{ij} + (GL)_{il} + (GYL)_{ijl} + \varepsilon_{ijkl} , $$

where *y*_*ijkl*_ is the mean yield of the *i*th genotype in the *j*th year, *k*th trial series and *l*th location, *μ* is the overall mean, *G*_*i*_ is the main effect of the *i*th genotype, *Y*_*j*_ is the main effect of the *j*th year, (*YT*)_*jk*_ is the *k*th trial series within the *j*th year, *L*_*l*_ is the *l*th location, (*YL*)_*jl*_ is the *jl*th year × location interaction effect, (*YTL*)_*jkl*_ is the *l*th location effect within the *k*th trial series in the *j*th year, (*GY*)_*ij*_ is the *ij*th genotype × year interaction effect, (*GL*)_*il*_ is the *il*th genotype × location interaction effect, $(GYL)_{ijl}$ is the *ijl*th genotype × year × location interaction effect, *ε*_*ijkl*_ is a residual error of adjusted means from a randomized block, split-plot or α-lattice design. For laboratory traits, errors also arise from laboratory tests of bulked grain samples. In the case of α-lattice design, we used adjusted means which may be correlated. We did not consider this correlation in our analysis, however, because a weighted analysis would have been computationally rather more demanding than the unweighted analysis we did here and because results between weighted and unweighted analyses are usually rather similar for the types of trials we consider here (Möhring and Piepho 2009). Our model assumes that trial series are nested within years and that locations are crossed with years, i.e., at least some locations are used across several years. As laboratory samples for grain and malting traits were analyzed by three different laboratories, but a full series was only analyzed by a single laboratory, the laboratory effect is confounded with the effect for trial series. All effects except *G*_*i*_ and *Y*_*j*_ are assumed to be random and independent with constant variance for each effect. Genetic and non-genetic time trend were studied by modeling *G*_*i*_ and *Y*_*j*_ with regression terms for time trends as follows (Piepho et al. 2014a; Laidig et al. 2014, 2017):

2 $$ G_{i} = \beta r_{i} + H_{i} , $$

where $\beta$ is a fixed regression coefficient for genetic trend, *r*_*i*_ is the first year of testing for the *i*th variety, and *H*_*i*_ models a random normal deviation of *G*_*i*_ from the genetic trend line, and

3 $$ Y_{j} = \gamma t_{j} + Z_{j} , $$

where $\gamma$ is a fixed regression coefficient for the non-genetic trend, *t*_*j*_ is the continuous covariate for the calendar year and *Z*_*j*_ is a random normal residual. Genetic and non-genetic trends are quantified by the regression coefficients *β* and *γ*, respectively, indicating the yield increase per year measured in the same units as *y*_*ijkl*_.

*Model for overall trend* Overall trend was modeled considering the genotype as nested within years (Laidig et al. 2014). Thus, compared with Model (1), for this analysis we dropped effects involving genotypes that are not nested within years, i.e., we dropped the effects $G_{i}$ and (*GL*)_*ij*_. The reduced model is given by:

4 $$ y_{ijkl} = \mu + Y_{j} + (YT)_{jk} + L_{l} + (YL)_{jl} + (YTL)_{jkl} + (GY)_{ij} + (GYL)_{ijl} + \varepsilon_{ijkl} . $$

Similarly as in Eq. (3), the year main effect can be modeled as:

5 $$ Y_{j} = \phi t_{j} + U_{j} , $$

where $\phi$ is a fixed regression coefficient for overall trend, *t*_*j*_ is the continuous covariate for the calendar year and *U*_*j*_ is a random residual following a normal distribution with zero mean and variance $\sigma_{U}^{2}$. We take the year main effects as fixed to obtain adjusted means for years, representing the overall trend.

*Genetic correlation* We estimated bivariate genetic correlation coefficients between traits based on three univariate analyses as described in Piepho et al. (2014b).

*Principal component analysis* To provide a first graphical look on the genetic correlation pattern, we carried out a principal component analysis with the correlation matrix as computed from the pairwise genetic correlation coefficients between traits *ρ*_g_ as input. We standardized the eigenvectors obtained by the spectral decomposition of the correlation matrix by multiplying them with the square root of the corresponding eigenvalue. Figure 5 shows the standardized component pattern of trait vectors plotted on the first two principal components. The cosine of the angle *α* between the vectors of two traits can be interpreted as an approximation of the genetic correlation coefficient *ρ*_g_ between both traits. If *α* is between 0° and 90°, then both traits are positively associated (0 < *ρ*_g_< 1), and if *α* is between 90° and 180°, then both traits are negatively associated (0 > *ρ*_g_ > −1) (Digby and Kempton 1987). We further plotted two circles with radius 1 and 0.707 centred at the origin of the coordinates. Trait vectors reaching the 100% (radius 1) circle indicate that their variability is represented completely by the first two principal components, and vectors reaching the 50% (radius 0.707) circle that their variability is represented by 50%. Generally, the length of a trait vector indicates how much of its variability is represented by the two principal components (Abdi and Williams 2010).

*Graphical displays* We define a fixed categorical effect $C_{d}$ for group $d = 1, \ldots ,D$, where $D$ is the number of levels of the time variable $r_{i}$, where each group is represented by at least one genotype. Then, the genetic effect can be modeled as:

6 $$ G_{i} = C_{d} + H_{i}^{\prime } , $$

where $H_{i}^{\prime }$ is the random deviation from categorical effect $C_{{_{d} }}$. We compute adjusted means for $C_{d}$ and plot them against first year of testing $r_{i}$ (Piepho et al. 2014a).

The following plots can be considered based on the proposed models:

1. Visible genetic trend: plot of adjusted genotype group means for $C_{d}$ based on (6), inserted in the baseline Model (1), against time ($r_{i}$).
2. Visible agronomic trend: plot of adjusted year means for $Y_{j}$ using the baseline Model (1) against calendar year *t*_*j*_.

## Results

### Performance progress

**Fig. 2:**
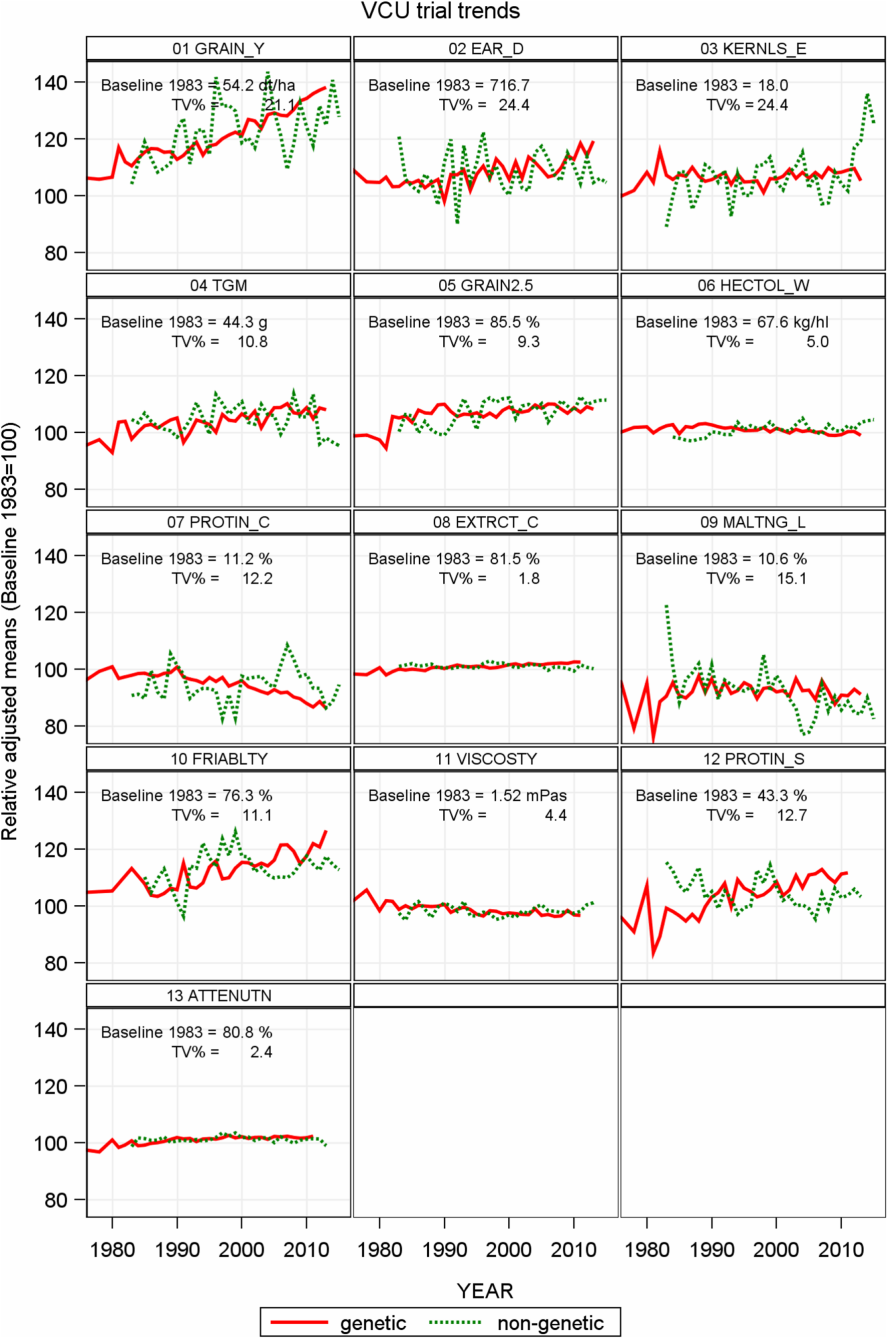
Relative adjusted means as percent of 1983 baseline (overall regression estimate 1983, Table 2). Genetic: variety group means [effect C_d_ in Eq. (6)]. Non-genetic: year means [Eq. (1), using Eq. (6) to model *G*_*i*_]. *TV%* total variation (square root of sum of variance components) as percent of 1983 baseline, *YEAR* varieties’ first year in trial *r*_*i*_ for genetic trend and calendar year *t*_*k*_ for non-genetic trend. *GRAIN_Y* grain yield at 86% dry matter, EAR*_D* single ear density, *KERNLS_E* number of kernels per ear, *TGM* thousand grain mass at 86% dry matter, *GRAIN2.5* grain fraction with kernel size >2.5 mm, *HECTOL_W* hectoliter weight (test weight), *PROTIN_C* crude grain protein concentration [% of dry matter], *EXTRCT_C* extract content in dry matter (%), *MALTNG_L* malting loss, *FRIABLTY* friability, *VISCOSTY* viscosity, *PROTIN_S* protein solution degree (Kolbach value), *ATTENUTN* final attenuation degree

**Fig. 3:**
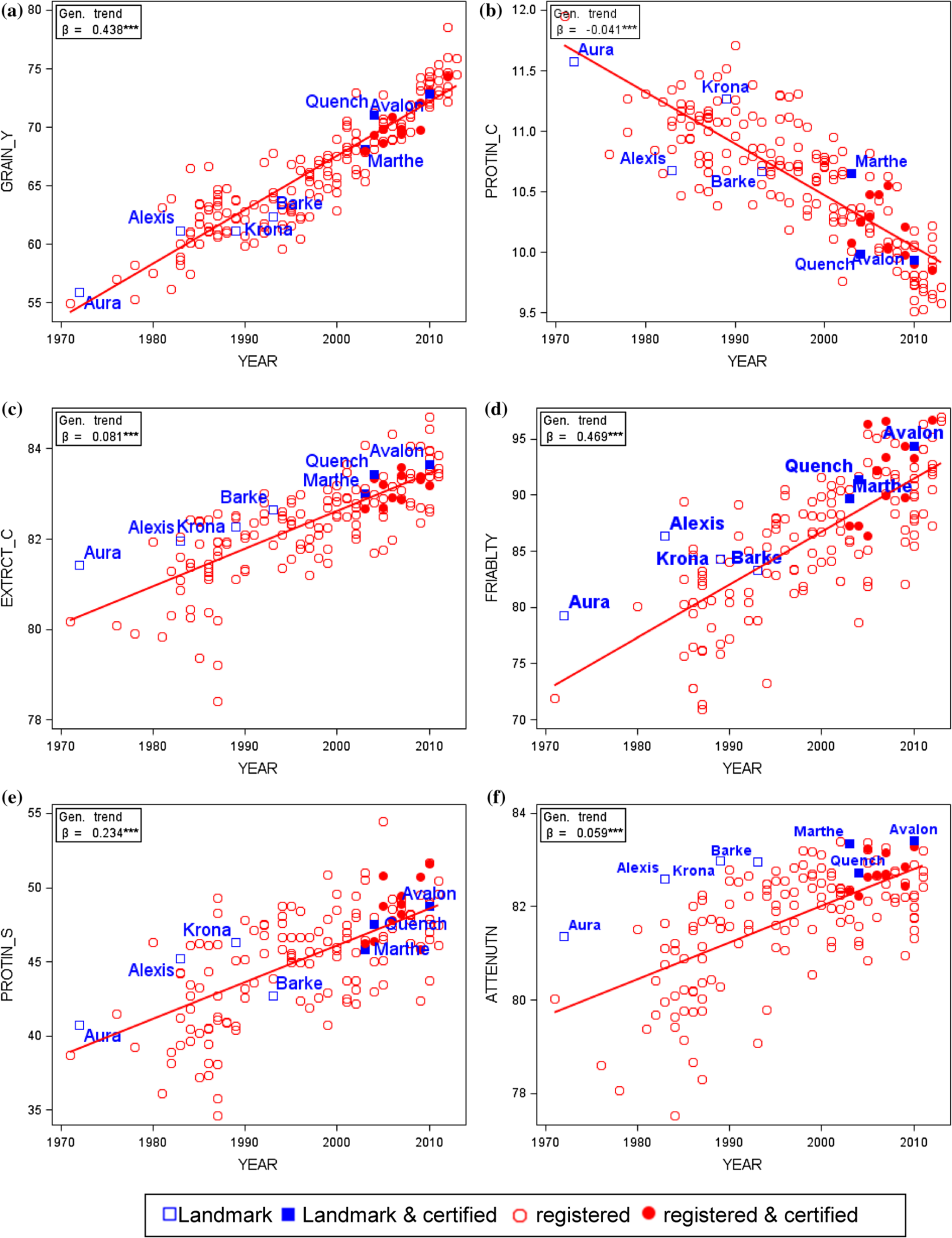
Adjusted variety means [Eq. (1) keeping effects of genotype *G*_*i*_ and years *Y*_*k*_ fixed] plotted against first year in trial. *YEAR* first year in trial, *GRAIN_Y* grain yield at 86% dry matter, *PROTIN_C* crude grain protein concentration (% of dry matter), *EXTRCT_C* extract content in dry matter (%), *FRIABLTY* friability, *PROTIN_S* protein solution degree (Kolbach value), *ATTENUTN* final attenuation degree. *Landmark* dominating variety, *certified* certified by German Brewing Barley Association, *registered* registered for VCU. *β* genetic trend [Eq. (1) using Eq. (2)]. *Significant at 5% level; **significant at 1% level; ***significant at 0.1% level

In Table 2, we compare trends representing progress achieved in VCU trials for 13 traits between 1983 and 2015. In Fig. 2, relative adjusted variety group and year means are displayed as percentage of 1983 baseline to make observed trends comparable between traits. The baseline is the predicted value in 1983 based on the overall regression trend. Simply speaking, the genetic trend line represents the progress contributed solely by new varieties, and the non-genetic trend line indicates the progress if no new varieties were introduced and only non-genetic factors generated change. In Fig. 3 and in Electronic Appendix Fig. S1, we plotted adjusted variety means against their first year in trials, highlighting six varieties (Alexis, Aura, Avalon, Barke, Marthe, Quench) as landmarks to give orientation and visualize genetic progress. These varieties were, and some still are, dominating with high brewing quality and are of widespread use. These six varieties were also included as standards in VCU trials. Varieties which were further tested in the “Berliner Programm” (Braugersten-Gemeinschaft 2016b) and certified by German Brewing Barley Association (Braugersten-Gemeinschaft e. V.) since 2005 are identified by blue and red filled symbols in Fig. 3 and in Electronic Appendix Fig. S1.

**Table 2:** Performance levels 1983 and 2015, difference in performance levels between 2015 and 1983 based on overall regression estimates, and estimates of linear regression coefficients for trends

Traits		Predicted values 1983 and 2015 based on overall linear trends					Estimates of linear trends											
							Genetic				Non-genetic				Overall			
Short name^a^	Unit	1983	2015	Diff.	Sign.	%Diff	Absolute	Sign.	SE	%	Absolute	Sign.	SE	%	Absolute	Sign.	SE	%
Yield components																		
GRAIN_Y	dt ha^−1^	54.2	77.6	23.4	***	43.0	0.438	***	0.026	0.81	0.294	***	0.092	0.54	0.729	***	0.088	1.34
GRAIN_Y^b^	dt ha^−1^	40.0	54.0	14.0	***	35.0									0.438	***	1.095	1.10
EAR_D	ears m^−2^	716.7	832.2	115.5	***	16.1	2.348	***	0.525	0.33	1.020		1.143	0.14	3.611	***	1.061	0.50
KERNLS_E	kernels ear^−1^	18.0	20.5	2.5	**	14.3	0.009		0.012	0.05	0.075	*	0.032	0.42	0.081	**	0.030	0.45
TGM	g (1000 kernels)^−1^	44.3	47.6	3.3	*	7.5	0.138	***	0.020	0.31	−0.029		0.044	−0.07	0.104	*	0.042	0.23
Grain quality																		
GRAIN2.5	%	85.5	97.0	11.5	***	13.5	0.121	***	0.033	0.14	0.222	***	0.060	0.26	0.361	***	0.051	0.42
HECTOL_W	kg hl^−1^	67.6	68.8	1.2		1.8	−0.062	***	0.012	−0.09	0.099	***	0.025	0.15	0.037		0.024	0.06
PROTIN_C	%	11.2	10.0	−1.2	**	−10.1	−0.041	***	0.004	−0.37	0.005		0.013	0.05	−0.035	**	0.013	−0.32
Malting quality																		
EXTRCT_C	%	81.5	83.3	1.8	***	2.3	0.081	***	0.008	0.10	−0.020		0.014	−0.03	0.057	***	0.013	0.07
MALTNG_L	%	10.6	8.9	−1.7	***	−16.7	−0.002	***	0.008	−0.02	−0.052	***	0.014	−0.49	−0.055	***	0.012	−0.52
FRIABLTY	%	76.3	95.5	19.2	***	25.1	0.469	***	0.049	0.62	0.136		0.097	0.18	0.599	***	0.088	0.79
VISCOSTY	mPas	1.52	1.46	−0.06	**	−4.2	−0.002	***	0.000	−0.14	0.000		0.001	0.01	−0.002	**	0.001	−0.13
PROTIN_S	%	43.3	47.4	4.1	**	9.3	0.234	***	0.031	0.54	−0.092		0.047	−0.21	0.126	**	0.041	0.29
ATTENUTN	%	80.8	83.2	2.4	***	3.0	0.059	***	0.011	0.07	0.017		0.019	0.02	0.077	***	0.019	0.10

Per cent trends (%) are relative to 1983 performance levels

*GRAIN_Y* grain yield at 86% dry matter, *EAR_D* single ear density, *KERNLS_E* number of kernels per ear, *TGM* thousand grain mass at 86% dry matter, *GRAIN2.5* grain fraction with kernel size >2.5 mm, *HECTOL_W* hectoliter weight (test weight), *PROTIN_C* crude grain protein concentration (% of dry matter), *EXTRCT_C* extract content in dry matter [%], *MALTNG_L* malting loss, *FRIABLTY* friability, *VISCOSTY* viscosity, *PROTIN_S* protein solution degree (Kolbach value), *ATTENUTN* final attenuation degree, *SE* standard errors of regression coefficients

* Significant at 5% level

** Significant at 1% level

*** Significant at 0.1% level

^a^For full name and description of trait see Table 1

^b^On-farm national average yield

#### Grain yield and yield components

Grain yield in VCU trials increased by 43% (23.4 dt ha^−1^) in the last 33 years relative to the 1983 yield level, corresponding to an average increase of 1.34% per year. Genetic and non-genetic causes contributed significantly to this considerable progress (Table 2). On-farm yield progress in Germany was at a lower level of 35% (14.0 dt ha^−1^) as compared to VCU trials. The gap between both trends even widened from 14.2 dt ha^−1^ in 1983 to 23.6 dt ha^−1^ in 2015 (Table 2; Fig. 1). All yield components contributed significantly to increasing barley yield, whereas ear density showed the largest increase of 16.1% (115.5 ears m^−2^), followed by kernels per ear of 14.3% (2.5 kernels ear^−1^) and thousand grain mass of 7.5% (3.3 g) relative to 1983. Genetic and non-genetic trends (β and γ) indicated that gain in thousand grain mass was completely, and ear density was mainly based on new varieties.

#### Grain quality

Our results indicated that the grain fraction with kernels >2.5 mm increased significantly by 13.5% (11.5% absolute change) in the last 33 years relative to 1983, both by genetic and non-genetic effects. For protein concentration we found a significant decline of 10.1% (−1.2% absolute change) relative to 1983 caused nearly completely by genetic effects (Fig. 3b). For hectoliter weight, the significantly negative genetic trend was overcompensated by a significantly positive non-genetic trend, resulting in a non-significant overall change of 1.8% (1.2% absolute change, Table 2).

#### Malting quality

Results in Table 2 show a significant improvement for all traits achieved by breeders and VCU testing during the last 33 years, however, with different magnitude as Fig. 2 shows. Friability of malt rose by 25.1% (19.2% absolute change) mainly due to genotypic effects, whereas malting loss could be reduced from 10.6% in 1983 to 8.9% in 2015, which is equivalent to −16.7% (−1.7% absolute change) relative to 1983, caused mainly by non-genetic effects. Extract content, the most important malting trait, showed the lowest increase of 2.3% (1.8% absolute change), however, highly significant and completely due to genetic improvement. Figure 3c, d highlight the outstanding performance of landmark varieties for extract content and final attenuation degree.

### Genotypic and environmental variation

**Fig. 4:**
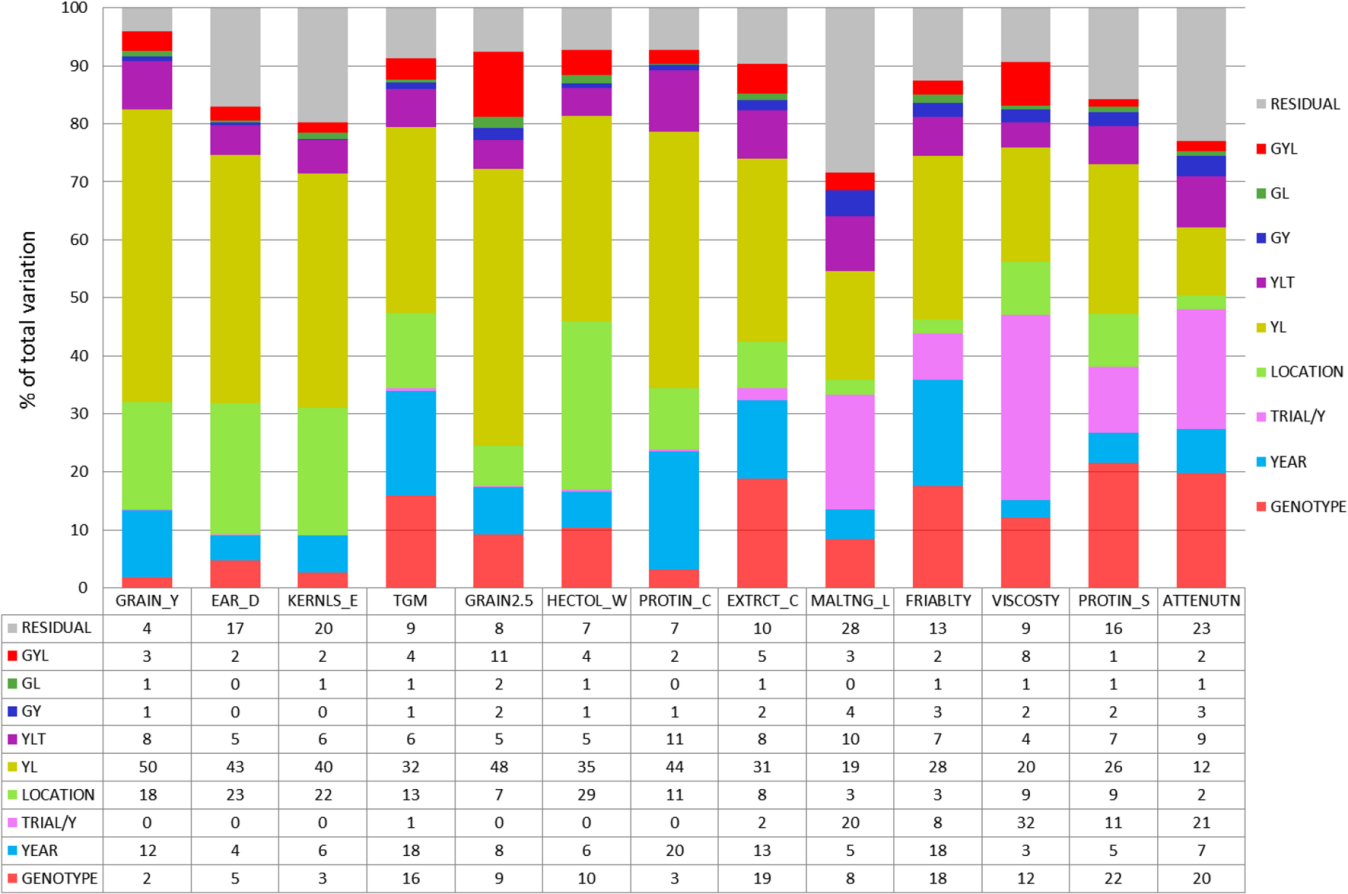
Sources of variation of traits from VCU trials after elimination of genetic and non-genetic trends as percentage of total variability [Eq. (1), using Eqs. (2) and (3)]. *GRAIN_Y* grain yield at 86% dry matter, *PLANT_H*, *EAR_D* single ear density, *KERNLS_E* number of kernels per ear, *TGM* thousand grain mass at 86% dry matter, *GRAIN2.5* grain fraction with kernel size >2.5 mm, *HECTOL_W* hectoliter weight (test weight), *PROTIN_C* crude grain protein concentration (% of dry matter), *EXTRCT_C* extract content in dry matter (%), *MALTNG_L* malting loss, *FRIABLTY* friability, *VISCOSTY* viscosity, *PROTIN_S* protein solution degree (Kolbach value), *ATTENUTN* final attenuation degree

As estimates of long-term variance components may be biased if time trends are present in random effects, we based our estimates on Model (1), taking into account linear trends which may be contained in genotypic and year effects. Variance components for the genotypic effect *H*_*i*_ [Model (2)] and the year effect *Z*_*j*_ [Model (3)] are then unbiased deviations from linear trends. It is useful and illustrative to express variance components as percentage of their total sum (Fig. 4). As explained in the “Materials and methods” section, we included in our Model (1) a term for trial series within the year main effect (*YT*)_*jk*_ and within the year × location interaction (*YTL*)_*jkl*_ to take into account variation caused by environmental differences between trial series, and additionally by different laboratories analyzing different series. Further, we derived a measure to quantify the relative total variation in percent (TV%) of an individual trait, calculated as the square root of the sum of the estimated variance components, expressed as percentage of the 1983 overall regression estimate as given in Table 2. Note that TV% is essentially a coefficient of variation. The relative total variation of individual traits is shown as insets in Fig. 2.

#### Grain yield and yield components

Genotypic and genotype × environmental variation of grain yield and yield components are very low within the range of 0–5%, except for thousand grain mass where genotypes account for more than 16% of total variation (Fig. 4). The major influence on variability is due to environmental factors. The location main effects are subject to a higher variability (13–23%) than years (4–18%).The component of trial series within years apparently accounted for no more than 1%, i.e., there were no differences between trial series for yield and yield components. The residual variation of grain yield (4%) is by far the smallest one of all 13 traits (Fig. 4). All traits are subject to high total variability with TV% above 20%, except for thousand grain mass with TV% = 10.8% (Fig. 2).

#### Grain quality

Genotypic and environmental variation of grain quality traits is similar as observed for yield and yield components (Fig. 4). For protein concentration, we found a remarkably high variation for the year main effect (20%) and for hectoliter weight a very high influence of location (29%). Residual error variances are below 10% of total variation. Hectoliter weight is very stable with a total variability of TV% = 5.0% (Fig. 2).

#### Malting quality

Variation of genotypes for malting traits is substantially larger than for other traits. Genotypic variation was highest for protein solution degree (22%) and lowest for malting loss (8%) (Fig. 4). In contrast to the traits for yield and its components, and for grain traits, the effect of trial series within years accounts for a large part of variation, except for extract content. For viscosity 32% of total variation is due to differences between trials within years indicating that the three laboratories contribute considerably to variation of malting traits. But the trial series effect within year × location interaction (4–10%) was of about the same magnitude as for other traits. Noticeably high residual error variances were observed for malting loss (28%) and final attenuation (23%). The low total variation of extract content (TV% = 1.8%) and final attenuation degree (TV% = 2.4%) indicate that malting traits are highly stable (Fig. 2).

## Phenotypic and genetic correlation

We calculated phenotypic correlation coefficients *ρ*_p_ between all 13 traits based on adjusted variety means and the genetic correlations coefficients *ρ*_g_ (Table 3). In contrast to phenotypic correlation, which depends on genotypes, time trends and environmental effects, genetic correlation can be considered as a measure of the association between two traits solely due to genotypes, controlling for time trend, locations and years. A genotypic correlation coefficient of zero indicates that the genetic effects between varieties are independent. In Fig. 6 and in Electronic Appendix Fig. S2, we plotted correlation diagrams of adjusted variety means, highlighted landmark varieties, and varieties certified for their processing quality by the German Barley Association since 2005 to highlight their superior malting quality. To give orientation in the correlation diagrams, we drew horizontal and vertical reference lines for each plot representing averages over adjusted variety means.

**Table 3:** Correlations

	GRAIN_Y	EAR_D	KERNLS_E	TGM	GRAIN2.5	HECTOL_W	PROTIN_C	EXTRCT_C	MALTNG_L	FRIABLTY	VISCOSTY	PROTIN_S	ATTENUTN
*N*	187	186	186	187	187	186	187	156	164	151	156	156	156
Mean	66.3	774.5	19.3	46.3	91.7	68.2	10.6	82.3	9.7	86.4	1.49	45.3	81.7
Min	55.0	672.6	16.7	40.1	80.8	64.1	9.5	78.5	8.0	70.7	1.43	34.8	77.6
Max	78.3	944.0	21.7	52.9	97.1	72.0	12.0	84.6	11.0	97.1	1.61	54.2	83.4
Max–min	23.3	271.4	5.1	12.8	16.3	7.9	2.5	6.1	3.0	26.4	0.18	19.4	5.8
Traits	Correlation coefficients *ρ*												
GRAIN_Y	1												
	1												
EAR_D	0.59	1											
	0.35	1											
KERNLS_E	0.16^ns^	−0.35	1										
	0.05^ns^	−0.46	1										
TGM	0.54	−0.05^ns^	−0.19	1									
	0.07^ns^	−0.54	−0.35	1									
GRAIN2.5	0.20	−0.12^ns^	−0.04^ns^	0.48	1								
	−0.45	−0.42	−0.13^ns^	0.36	1								
HECTOL_W	−0.50	−0.26	−0.13^ns^	−0.29	0.04^ns^	1							
	−0.26	0.00^ns^	−0.02^ns^	−0.08^ns^	0.23	1							
PROTIN_C	−0.88	−0.55	−0.15^ns^	−0.44	−0.09^ns^	0.58	1						
	−0.56	−0.26	−0.13^ns^	0.06^ns^	0.45	0.56	1						
EXTRCT_C	0.61	0.38	0.04^ns^	0.35	0.39	−0.30	−0.62	1					
	−0.57	0.02^ns^	0.27	−0.23	0.16^ns^	0.09^ns^	0.02^ns^	1					
MALTNG_L	−0.09^ns^	0.05^ns^	0.12^ns^	−0.29	0.17^ns^	0.00^ns^	0.06^ns^	0.31	1				
	−0.47	0.06^ns^	0.21	−0.48	0.13^ns^	−0.01^ns^	0.06^ns^	0.50	1				
FRIABLTY	0.66	0.42	0.06^ns^	0.37	0.28	−0.50	−0.66	0.71	0.22	1			
	−0.34	−0.02^ns^	0.10^ns^	−0.16^ns^	0.10^ns^	−0.25	−0.15^ns^	0.38	0.41	1			
VISCOSTY	−0.52	−0.35	−0.09^ns^	−0.24	−0.25	0.34	0.45	−0.72	−0.38	−0.82	1		
	0.30	−0.03^ns^	0.00^ns^	0.25	−0.03^ns^	0.11^ns^	−0.11^ns^	−0.58	−0.52	−0.81	1		
PROTIN_S	0.50	0.33	−0.03^ns^	0.29	0.29	−0.38	−0.53	0.76	0.38	0.73	−0.70	1	
	−0.46	0.01^ns^	−0.11^ns^	−0.15^ns^	0.03^ns^	−0.15^ns^	−0.05^ns^	0.56	0.52	0.57	−0.57	1	
ATTENUTN	0.43	0.18^ns^	0.06^ns^	0.28	0.34	−0.14^ns^	−0.40	0.64	0.26	0.63	−0.72	0.60	1
	−0.36	−0.12^ns^	0.01^ns^	−0.12^ns^	0.13^ns^	0.21	0.19^ns^	0.37	0.35	0.48	−0.59	0.26	1

Categorization: 0.25 < |*ρ*| < 0.45 weak, 0.45 ≤ |*ρ*| < 0.65 moderate, 0.65 ≤ |*ρ*| < 0.85 strong, 0.85 ≤ |*ρ*| very strong

*N* number of varieties, *Mean* average over variety means, *Min* smallest variety mean, *Max* largest variety mean, *Upper value* phenotypic correlation coefficient *ρ*_p_, *Lower value* genetic correlation coefficient *ρ*_g_, *GRAIN_Y* grain yield at 86% dry matter, *EAR_D* single ear density, *KERNLS_E* number of kernels per ear, *TGM* thousand grain mass at 86% dry matter, *GRAIN2.5* grain fraction with kernel size >2.5 mm, *HECTOL_W* hectoliter weight (test weight), *PROTIN_C* crude grain protein concentration (% of dry matter), *EXTRCT_C* extract content in dry matter (%), *MALTNG_L* malting loss, *FRIABLTY* friability, *VISCOSTY* viscosity, *PROTIN_S* protein solution degree (Kolbach value), *ATTENUTN* final attenuation degree

^ns^Not significant different from zero at 1% level

### Principal components

**Fig. 5:**
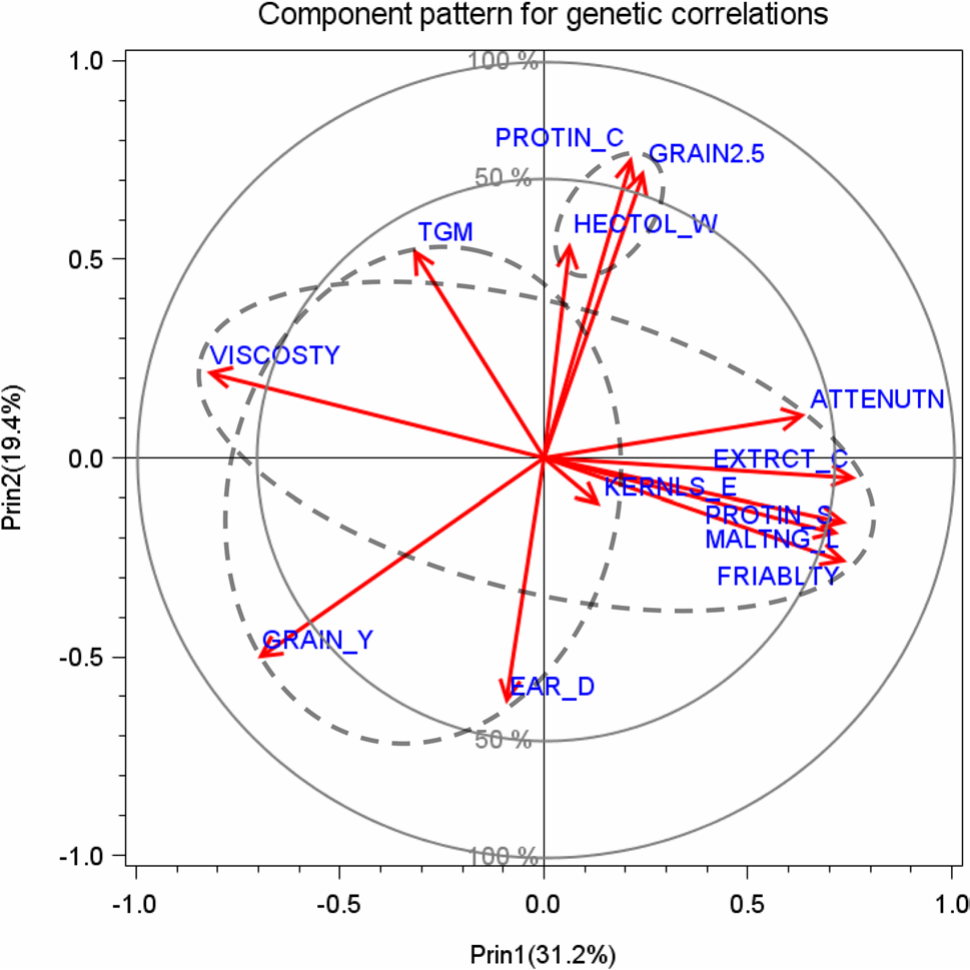
Component pattern for principal components 1 and 2 for genetic correlations of grain yield and components, grain traits, and malting traits (*dashed ellipsis*). *Unity circle* (100%) indicates that variation of traits is explained fully and the *smaller circle* (50%) that only half is explained by principle components 1 and 2, respectively. *GRAIN_Y* grain yield at 86% dry matter, *EAR_D* single ear density, *KERNLS_E* number of kernels per ear, *TGM* thousand grain mass at 86% dry matter, *GRAIN2.5* grain fraction with kernel size >2.5 mm, *HECTOL_W* hectoliter weight (test weight), *PROTIN_C* crude grain protein concentration (% of dry matter), *EXTRCT_C* extract content in dry matter (%), *MALTNG_L* malting loss, *FRIABLTY* friability, *VISCOSTY* viscosity, *PROTIN_S* protein solution degree (Kolbach value), *ATTENUTN* final attenuation degree

The association between traits represented by the first and second principal component is depicted in Fig. 5. Both components explained about 50% of total variation. Three different groups of traits can be seen in Fig. 5. The first group is composed of grain yield and its components, the second of grain traits and the third of malting traits. The first principal component (*x*-axis) is mainly spanned by traits of the third group, i.e., final attenuation degree, extract content, malting loss and friability on the positive side of the scale, balanced by viscosity on the negative side. Traits located on the positive side of the *x*-axis in this group are positively correlated with one another but negatively with viscosity which is located on the negative side of the *x*-axis. In other words, the first principal component can be referred to as a factor mainly related to malting quality. The spread along the second principal axis is determined by the second group, i.e., the grain traits on the positive side, and by two traits of the first group, i.e., grain yield and ear density on the negative side of the *y*-axis. The relative proximity of malting traits and their nearly orthogonal position relative to grain traits indicate that malting traits are interrelated, but only weakly associated with grain traits (Fig. 5). Vectors of grain yield and ear density point in the opposite direction to grain traits demonstrating a mainly negative association. Angles between grain yield and malting traits, which are between 90° and 180°, suggest a negative association, except for viscosity. The vector of kernels per ear is short which means that for this trait only a small proportion of variability is explained by principal components 1 and 2, and that this trait shows no or only a weak relationship with other traits.

### Comparing phenotypic and genetic correlation coefficients

**Fig. 6:**
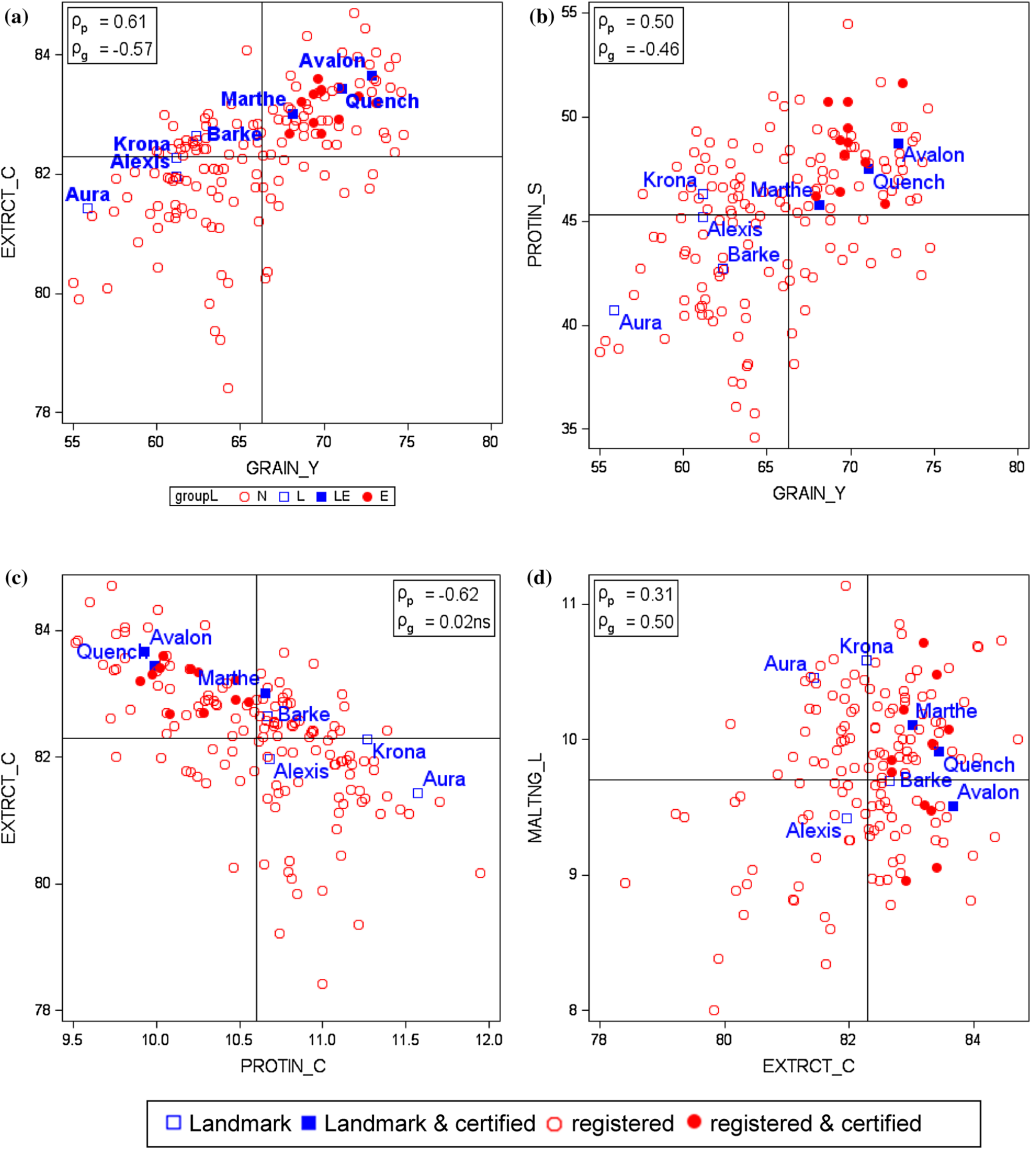
Phenotypic correlation of adjusted variety means [Eq. (1) keeping effects of genotype *G*_*i*_ and years *Y*_*k*_ fixed]. *Reference lines* are the averages of adjusted variety means as given in Table 3. *ρ*_p_: phenotypic correlation coefficient; *ρ*_g_: genetic correlation coefficient. *ns* not significant different from zero if *p* > 0.01. *GRAIN_Y* grain yield at 86% dry matter, *PROTIN_C* crude grain protein concentration (% of dry matter), *EXTRCT_C* extract content in dry matter (%), *MALTNG_L* malting loss, *PROTIN_S* protein solution degree (Kolbach value). *Landmark* dominating variety, *certified* certified by German Brewing Barley Association, *registered* registered for VCU

When comparing the magnitude of phenotypic correlation coefficients with their corresponding genetic values in Table 3, we find considerable deviations, even with partially reversed signs, especially for coefficients of grain yield, thousand grain mass and protein concentration with other traits. For example, the phenotypic correlation between grain yield and extract content is *ρ*_p_ = 0.61, whereas the genetic correlation is *ρ*_g_ = −0.57 (Fig. 6a). For grain yield and viscosity, the correlations are *ρ*_p_ = −0.52 and *ρ*_g_ = 0.30 (Table 3). Correlation coefficients among malting traits and correlation coefficients for kernels per ear and hectoliter weight with other traits show good agreement between phenotypic and genetic coefficients (Fig. 6d, Electronic Appendix Figs. S2a–d).When considering the genetic correlations in Table 3, we can see that (1) kernels ear^−1^ and ear density, the three grain traits and the six traits for malting quality are relatively uncorrelated, (2) malting traits are moderately to strongly positively interrelated, but not so viscosity which is negatively correlated with the other malting traits, and (3) grain yield is weakly to moderately negatively correlated with grain and quality traits, but positively with viscosity.

## Discussion

### Performance progress

#### Grain yield and yield components

Grain yield is the most important trait for evaluating breeding progress because it finally determines the farmers’ revenue. We found an annual overall linear increase of 0.729 dt ha^−1^ between 1983 and 2015. Thereof, the genetic trend was about 60% (0.438 dt ha^−1^ year^−1^). Compared with results from six recent European spring barley studies (Baumer et al. 2000; Peltonen-Sainio et al. 2009; Psota et al. 2009; Grausgruber et al. 2002; Mackay et al. 2011; Rijk et al. 2013) on breeding progress between 1950 and 2010, with trends between 0.28 and 0.60 dt ha^−1^, and an average of 0.52 dt ha^−1^, our result of 0.44 dt ha^−1^ falls behind. A widening yield gap between VCU and on-farm grain with yields trends of 0.73 and 0.44 dt ha^−1^ year^−1^ for the two systems was observed (Table 2; Fig. 1), which is considerably wider and at a lower level than results from a Dutch study (Rijk et al. 2013), where progress in variety trials reached 0.9 and on-farm 0.7 dt ha^−1^ year^−1^, respectively. That farm yield level is below that of trials is not surprising. According to Cassman and Harwood (1995) and van Wart et al. (2013), grain yields at the farm scale could in general approach 75–85% of the genetic yield potential. One reason for the yield gap observed in this study may be that growers cannot use the full yield potential since they must balance application of nitrogen to increase yield and maintain moderate grain protein levels because brewing quality grain lots should have protein concentration below 11.5%. As mentioned earlier, the average price difference between brewing and fodder quality is currently more than 3 € dt^−1^. Therefore, it is more economical for growers to aim crop management at quality than at quantity. Furthermore, the considerable reduction of growing area for spring barley during the last 30 years in Germany (Fig. 1) could be another reason for a widening yield gap, because on fertile soils previously grown crops were partly replaced by more profitable crops, like winter barley or forage maize (Rijk et al. 2013).

Our results demonstrated that all yield components significantly contributed to yield progress. Whereas progress of ear density is mainly caused by new varieties, and thousand grain mass totally so, for kernels per ear only non-genetic progress was found (Table 2). Increasing seed rate can definitely be excluded as a reason for increasing ear density, because seed rates have been significantly reduced from 347 to 320 kernels m^−2^ during the studied period (Electronic Appendix Table S3). The strongly increasing genetic trend for thousand grain mass may partially be caused by an indirect selection effect on grain size in early breeding stages, which is supported by the positive genetic correlation of thousand grain mass and hectoliter weight (Table 3). In a study of Psota et al. (2009) in the Czech Republic including 99 spring barley varieties, an increase of thousand grain mass of 0.177 g year^−1^ between 1955 and 2005 was found, which was considerably higher than our result (0.138 g year^−1^). We further found that sowing dates were 4.5 days and harvest dates 5 days earlier in 2013 than at the beginning of our study period; however, these results were not significant (Electronic Appendix Table S3). Earlier sowing dates could likely have a favorable effect on non-genetically generated yield progress due to a more efficient use of spring moisture. Furthermore, the significant increase of annual average daily temperatures by more than 1 °C during the studied period (Electronic Appendix Table S3) may additionally have contributed to non-genetic yield progress. Further factors had a significant influence on genetic yield progress. As increasing plant density raised susceptibility of plants to leaf diseases, with mildew being the most important one, breeding for higher resistance was successfully intensified (Baumer et al. 2004; Grausgruber et al. 2002). We found a decreasing susceptibility for mildew of 1.3 susceptibility-score units observed on a 1–9 scale (3 slightly, 5 medium, 7 strongly susceptible). Baumer et al. (2004) reported that the number of varieties with resistance above average level increased from 20% in 1983 to 70% in 2003. Another positive effect on yield progress was achieved by a further reduction of plant height. In our study plant height declined by about 0.14 cm year^−1^ (data not shown in Tables). Ortiz et al. (2002) observed a reduction of plant height in Nordic spring barley varieties of 0.20 cm year^−1^ during 1948 and 1988. Smaller varieties are less prone to lodging, and have higher yield stability and a better grain quality.

#### Grain and malting traits

The grain fraction with kernels >2.5 mm increased considerably due to genetic and non-genetic effects. This grain fraction accounted for an estimated 97% of total grain in 2015 (Table 2). It seems unlikely that this trait can be further improved. The decline in protein concentration is not surprising, because it is generally known that a negative relationship exists between grain yield and protein concentration (e.g., Oberforster and Werteker 2011). This negative relation is in favor of good malting quality in contrast to baking quality of winter wheat where both traits are antagonistic (Laidig et al. 2017).

Our results in Table 2 and Fig. 2 evidenced a consistent significant improvement for all six malting traits mainly due to genetic factors. This is in agreement with numerous studies on breeding progress for malting barley (Gothard et al. 1983; Wych and Rasmusson 1983; Ogushi et al. 2002; Grausgruber et al. 2002; Passarella et al. 2002; Baumer et al. 2004). Our results, shown in brackets, are comparable with those found by Psota et al. (2009) from Czech trials for extract content with a value of 0.0641% (compared to 0.081% in our analysis), a protein solution degree of 0.146% (0.234%) and a final attenuation degree of 0.076% (0.059%). It is noticeable that the gain of extract content of 2.3% relative to 1983 was smallest among all 13 traits. This low gain is in agreement with, for example, Passarella et al. (2002) who state that in biological terms this increase is a small change though it is an important increase for brewing industry. Extract content has been a main breeding objective for a long time. Enormous progress was achieved in the years prior to our study period (e.g., Fischbeck et al. 2008; Psota et al. 2009). Baumer et al. (2000) reported an increase of extract content from 81.1% in 1900 to 84.3% in 2000, assuming at the time that no further increase for hulled barley would be possible (Baumer et al. 2004).

As mentioned in “Materials and methods”, the malting time was reduced by 1 day in 2002. To investigate whether this change affected malting traits, we extended our model for estimating genetic and non-genetic trends [Model (1) using Eqs. 2 and 3] by a fixed model term “period” representing a time effect for the study period until 2001 and one for the period after reduction of malting time in 2002. We further allowed for an interaction term for period with genetic and non-genetic trends to test if there was a deviation from the common genetic and non-genetic trends in both time periods. For all malting traits, our results did not indicate a significant deviation of genetic and non-genetic trends in both periods due to reduction of malting time in 2002 (data not shown in Tables). This result is supported by a graphical comparison of adjusted variety group means for malting traits, for grain yield and protein concentration (both were not influenced by change of malting time) against first year in trial, and for the adjusted year means against calendar year for the same traits (Electronic Appendix Fig. S3).

Generally, our results have shown that in barley breeding significant progress could be achieved in grain yield and malting quality despite the observed moderate negative genetic correlation, especially between grain yield and extract content (*ρ*_g_ = −0.57).

## Genotypic and environmental variation

The variance component estimates of this study are long-term estimates based on 33 years, more than 150 varieties and 70 different locations across Germany. In the literature, results from several studies on the influence of genotypes and environment on variation of agronomic and malting traits in barley have been reported (Rasmusson and Glass 1967; Rutger et al. 1966, 1967; Wych and Rasmusson 1983; Molina-Cano et al. 1997; Ogushi et al. 2002; Bertholdsson 2004; Condon et al. 2009), however, with only small numbers of varieties and/or environments which make results hardly comparable with our long-term results.

Figure 2 shows that total variability (TV%) is very different between traits; it may be as low as 1.8% for extract content and as high as 24.4% for ear density. Total variability of protein concentration is of medium variability (TV% = 12.2%); however, genotypic variation accounts for only 3% of total variation, indicating that environment and crop management, particularly nitrogen supply, are mainly responsible for variation of protein level in grain (Eagles et al. 1995). In contrast to protein concentration, extract content is a very stable trait with respect to total variability (TV% = 1.8%), however, with a relatively high genotypic share of 19% relative to total variation (Fig. 2). Figure 4 shows that at most 22% (protein solution degree) of total variation is due to genotypes whereas up to 98% (grain yield) is attributable to environment and genotype × environment interaction. Generally, our results agree with those of Rutger et al. (1966) in that variation of genotypic effects is higher for malting than for agronomic traits.

A substantial variance of the trial-within-year effect was observed for malting traits, except for extract content. In contrast, no such effects appeared for non-malting traits indicating that results between trial series are similar. As malting quality is tested in three different laboratories, but within a year each series is analyzed by only one laboratory, this discrepancy in variance component estimates for trial-within-year effects can probably be explained by laboratory effects. For extract content results of laboratories are apparently not that different. Laboratory effects should not alter rankings of varieties, because all quality samples of a complete trial series within a year are tested by the same laboratory.

We checked whether the shortening of the malting period influenced variation of malting traits by comparing the magnitude of the variance components as shown in Table 3 with those estimated by the extended model taking into account time period since 2001 and since 2002 as described in the previous section. The results showed that pattern of variance components from both models are very similar (data not shown).

## Phenotypic and genetic correlation

When considering phenotypic correlations between traits of this study based on adjusted variety means, we should be aware of associations due to different causes: (1) simultaneous selection of genetically independent traits could result in a correlation arising among those traits. (2) The traits could be controlled by the same genes or the traits could be components of each other. (3) The traits could be genetically linked and therefore selection for the one leads to indirect selection for the other (Condon et al. 2009).

For genetic correlations estimated in this study, dependencies arising from trends due to selection of genetically independent traits were eliminated by applying models including genetic trend, as described in “Statistical analysis” [Eqs. (2) and (3) used in Model (1)]. But for genetically dependent traits, correlation may be removed only partially from phenotypic correlation as far as it arises from a linear shift due to selection between 1983 and 2015. This applies especially for the phenotypic correlations for grain yield and protein concentration with other traits, which is largely due to genetic trends, however, not for phenotypic correlation of grain yield with protein concentration, because for cereal grains this relation has a genetic basis (e.g., Simmonds 1995; Hartl et al. 2011; Oberforster and Werteker 2011). Association between both traits, expressed by the genetic correlation (*ρ*_g_ = −0.56), is still negative but less stringent than the phenotypic coefficient (*ρ*_p_ = −0.87) (Table 3). Matthies et al. (2014) found an even stronger phenotypic correlation between grain yield and protein concentration (*ρ*_p_ = −0.91). A comparison between phenotypic and genetic correlation coefficients for extract content and other malting traits reveals similar effects (Table 3). Among these traits, the strongest correlation was observed between friability and viscosity (*ρ*_p_ = −0.82, *ρ*_g_ = −0.81), because friability is a strong indicator for cytolytic malt solution. Our overall finding, that correlation among malting traits was higher than among other traits, is in contrast to findings of Matthies et al. (2014), who stated that correlation between malting traits and agronomic traits is in general low indicating a genetic independence; however, we found some moderate to strong negative genetic correlations between grain yield and malting quality, for example between grain yield and extract content (*ρ*_g_ = −0.57), and protein solution degree (*ρ*_g_ = −0.46) (Table 3). The question arises if the change of malting time in 2002 could have influenced the relationship between grain yield and malting traits. To check this question, we estimated genetic correlation coefficients based on the extended model considering the period before and after change as described before. The results confirmed genetic correlations as shown in Table 3, not only for grain yield between malting traits, but also among malting traits (Electronic Appendix Table S4).

The strong negative phenotypic relation between protein concentration and extract content shown in Fig. 6c is in line with Arends et al. (1995) and Matthies et al. (2014), who both suggested that breeders should take into account this negative relation. But we found that both traits were not genetically correlated. In fact, the observed negative phenotypic correlation between protein concentration and extract content arises partially from selection for higher grain yield, which is genetically negatively associated with protein concentration, and simultaneous selection for higher extract content. Additionally, the observed negative phenotypic relation between grain protein concentration and extract content can be partially caused by environmental effects, i.e., environmental correlation, for example, due to varying nitrogen supply changing protein level in grain (Eagles et al. 1995). In consequence, both traits are probably not genetically related to each other, and hence indirect selection is not an option.

Grain size is generally used as an easily visually assessed selection trait in early stages of a breeding cycle. Despite of the weak phenotypic correlations with malting traits (−0.25 < *ρ*_p_< 0.39), no significant genetic correlations were found in our study (Table 3; Fig. 6), thus grain fraction with kernels >2.5 mm cannot be considered as a reliable indicator for malting quality. Our results are not in line with Bertholdsson (2004) who justified the use of grain fractions with kernel size >2.5 and >2.8 as an early selection criterion as they would show a certain degree of correlation with extract content.

Rutger et al. (1967) evaluated phenotypic and genetic correlations between grain yield and 9 malting traits and found that grain yield exhibited no really large correlations with other traits, whereas we found significant negative genetic correlations (−0.57 < *ρ*_g_ < −0.34) (Table 3). They further reported that it should be possible to select for most quality traits without seriously reducing yield. Our results confirmed that barley breeding achieved considerable progress in grain yield and malting quality (Table 2), despite of negative correlations between grain yield and malting traits.

## Conclusions

The current study is a baseline evaluation of the quantitative performance progress in spring barley, of variation and correlation of important yield and quality traits. The used large phenotypic data set covered a long time period, a wide range of different environments and a large collection of genotypes allowing statistically sound estimates and conclusions representative of Germany. We found significant overall gains for all traits, mainly due to genetic effects, which indicated effective breeding towards higher yield as well as improved malting quality. A widening gap between progress in trials and on-farm grain yield became apparent. We compared total variability between traits and found large variability for grain yield and relatively low ones for malting traits, especially for extract content and final attenuation degree. Estimates of variance components showed that genotypic variation of grain yield and protein concentration is very low as compared to malting traits. Our results illustrated that for long-term studies correlations between traits may be considerably influenced by selection. Hence, conclusions for breeding should be based on genetic rather than phenotypic correlation coefficients. In this study, we found no negative genetic correlation of protein concentration with malting quality, but between grain yield and malting quality. This negative association had apparently no visible effect on progress in malting quality. Our evaluation demonstrated the usefulness and cost-effectiveness of historic data analysis in reliably quantifying breeding progress and environmental impact on important traits.

### Author contribution statement

FL conceived the study, carried out the analyses, prepared the figures and tables and wrote the manuscript. HPP provided advice on statistical analysis, DR in using and interpreting data. Both read and amended the paper. TD and UM assembled all datasets, prepared and formatted them for statistical analysis. Both participated in editing the paper.

## Electronic supplementary material

Below is the link to the electronic supplementary material.

Supplementary material 1 (DOCX 158 kb)

Supplementary material 2 (DOCX 67 kb)

Supplementary material 3 (DOCX 77 kb)

Supplementary material 4 (DOCX 18 kb)

Supplementary material 5 (DOCX 24 kb)

Supplementary material 6 (DOCX 32 kb)

Supplementary material 7 (DOCX 23 kb)
