# Supplementary material for: Breeding progress, genotypic and environmental variation and correlation of quality traits in malting barley in German official variety trials between 1983 and 2015
Source: Theor Appl Genet. 2017 Aug 18;130(11):2411–29. doi: 10.1007/s00122-017-2967-4 (PMC5641284; doi:10.1007/s00122-017-2967-4)
Supplement: Supplementary file 1 — Supplementary material 1 (DOCX 158 kb) [file 122_2017_2967_MOESM1_ESM.docx]

(a) (b)


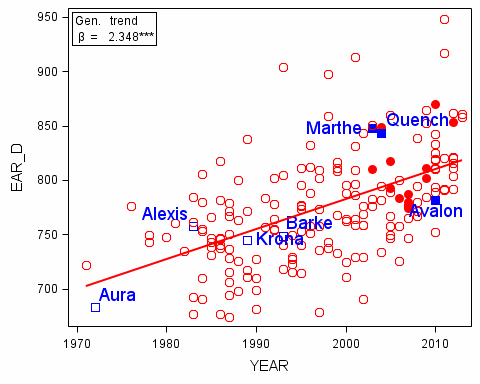

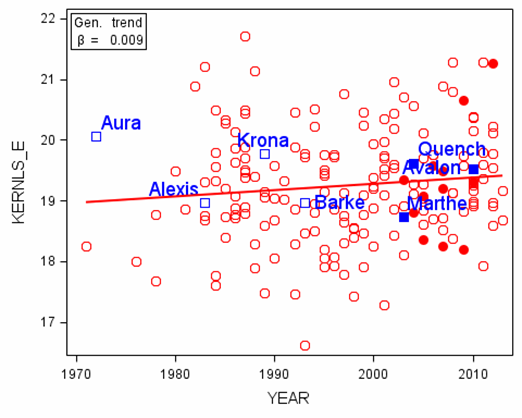


(c) (d)


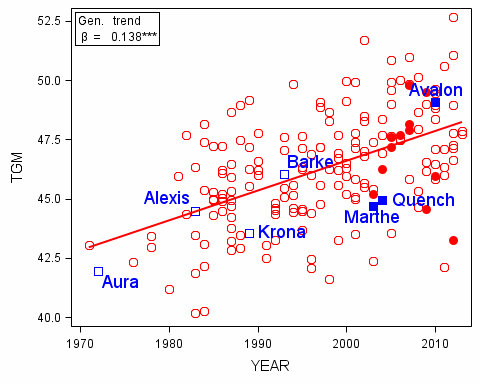

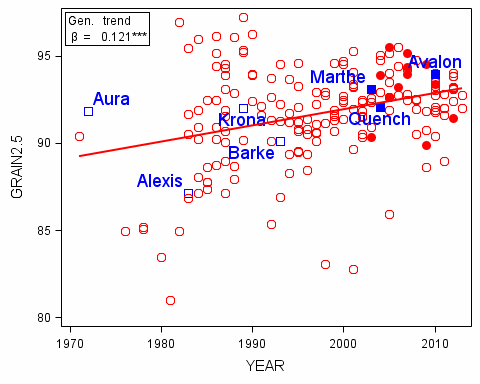


(e) (f)


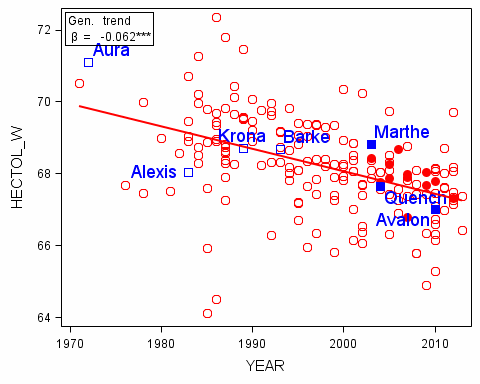

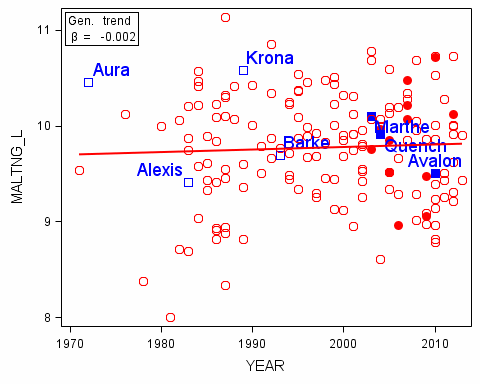


(g)


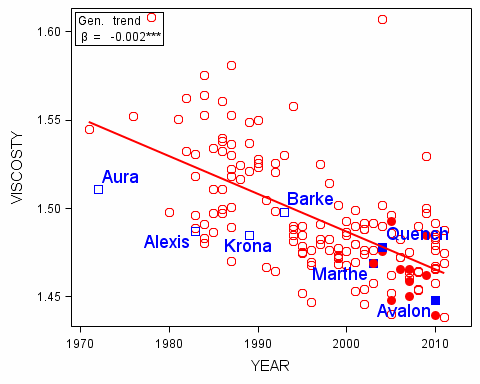


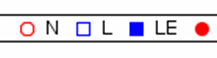
 Landmark
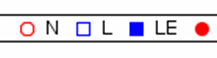
 Landmark & certified
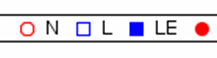
 registered
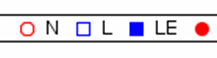
 registered & certified

**Fig. S1** Adjusted variety means [effect *G_i_* in Eq. (1)] plotted against first year in trial.

*EAR_D* Single ear density, *KERNLS_E* Number of kernels per ear, *TGM* Thousand grain mass at 86 % dry matter, *GRAIN2.*5 Grain fraction with kernel size > 2.5 mm, *HECTOL_W* Hectoliter weight (test weight), *MALTNG_L* Malting loss, *VISCOSTY* Viscosity

*Landmark* Dominating variety, *certified* Certified by German Brewing Barley Association, *registered* Registered for VCU

β: genetic trend [ Eq. (1) using Eq. (2)]

* significant at 5% level; ** significant at 1% level; *** significant at 0.1% level
