# Supplementary material for: Breeding progress, genotypic and environmental variation and correlation of quality traits in malting barley in German official variety trials between 1983 and 2015
Source: Theor Appl Genet. 2017 Aug 18;130(11):2411–29. doi: 10.1007/s00122-017-2967-4 (PMC5641284; doi:10.1007/s00122-017-2967-4)
Supplement: Supplementary file 2 — Supplementary material 2 (DOCX 67 kb) [file 122_2017_2967_MOESM2_ESM.docx]

(a) (b)


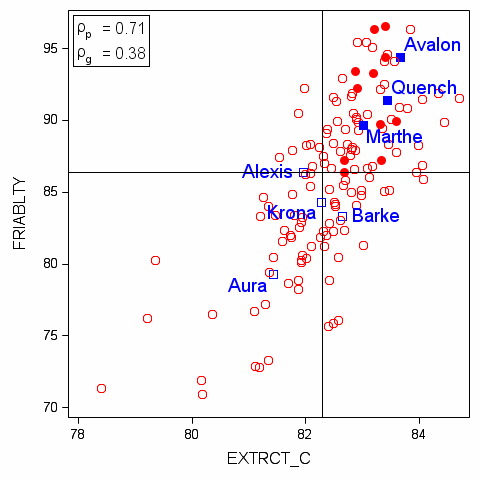

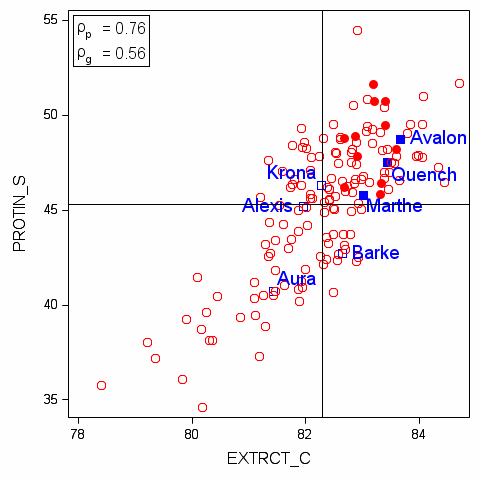


(c) (d)


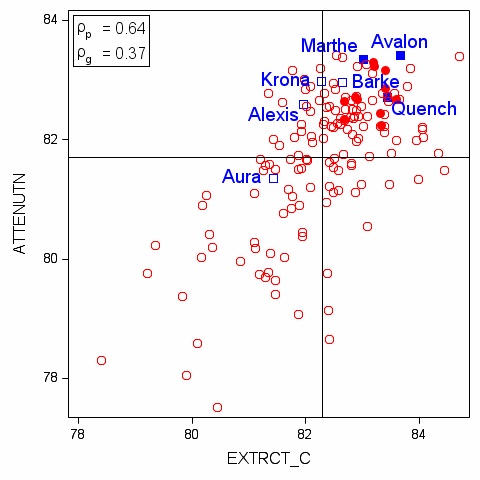

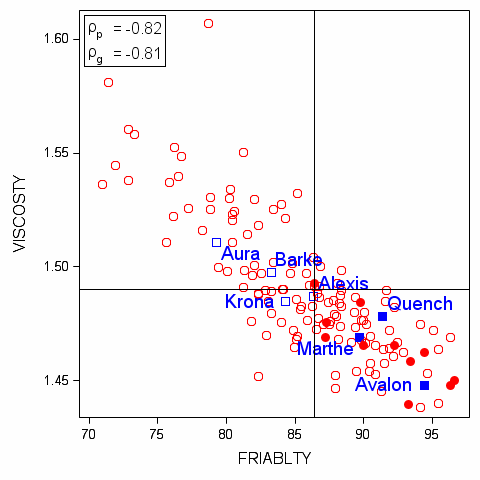


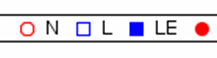
 Landmark
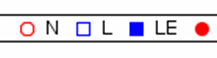
 Landmark & certified
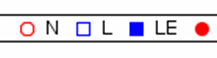
 registered
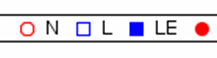
 registered & certified

**Fig. S2** Phenotypic correlation of adjusted variety means [Eq. (1) keeping effects of genotype *G_i_* and years *Y_k_* fixed]

Reference lines are the averages of adjusted variety means as given in Table 3.

ρ_p_: phenotypic correlation coefficient; ρ_g_: genetic correlation coefficient

*ns* not significant different from zero if p>0.01

*EXTRCT_C* Extract content in dry matter [%], *FRIABLTY* Friability, *VISCOSTY* Viscosity, *PROTIN_S* Protein solution degree (Kolbach value), *ATTENUTN* Final attenuation degree

*Landmark* Dominating variety, *certified* Certified by German Brewing Barley Association, *registered* Registered for VCU
