# Supplementary material for: Breeding progress, genotypic and environmental variation and correlation of quality traits in malting barley in German official variety trials between 1983 and 2015
Source: Theor Appl Genet. 2017 Aug 18;130(11):2411–29. doi: 10.1007/s00122-017-2967-4 (PMC5641284; doi:10.1007/s00122-017-2967-4)

**Reduction of Malting time from seven to six days in year 2002**

Beginning with harvest year 2002 malting time was reduced for all trial series from seven to six days, which may have affected all malting traits. This change allowed savings in time during the malting process due to varieties with higher diastatic power.

In addition to the check by the extended model, as described in Discussion, which did not indicate that trends changed in 2002, we plotted adjusted variety group-means for malting traits, and additionally for grain yield and protein concentration (both were not influenced by change of malting time) against first year in trial (Fig. S3a), and the adjusted year means against calendar year for the same traits (Fig.S3b). The curves for genetic trends do not indicate a change from 2002 on. Curves of traits for non-genetic trends show large fluctuations from year to year except for extract content, viscosity and final attenuation. The malting traits did not show a clear picture of a change due to a shorter malting time. The plots support our results from model check that trends were not significantly influenced by change of malting time.

(a)


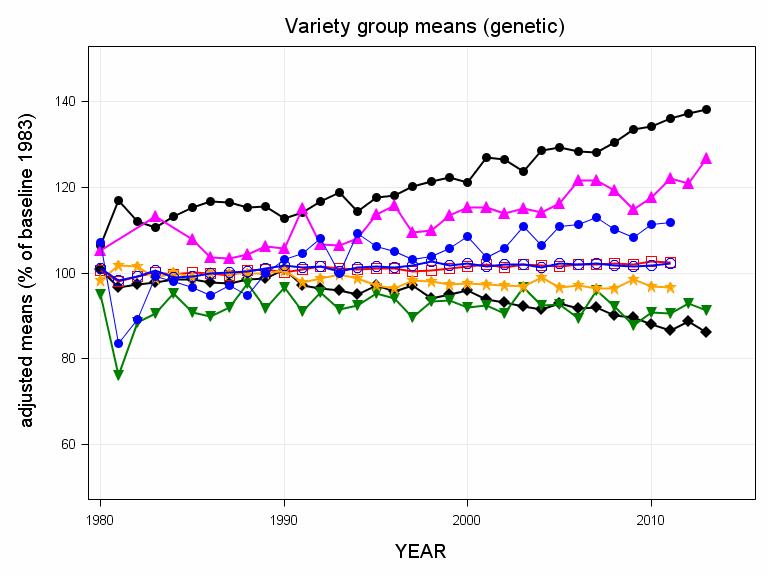


**Fig. S3** Relative adjusted means as percent of 1983 baseline (Overall regression estimate 1983, Table 2).

(a) Genetic: variety group means [effect *C_d_* in Eq. (6)]. (b) Non-genetic: year means [Eq. (1), using Eq. (6) to model *G_i_*].

*YEAR* (a) varieties’ first year in trial *r_i_* for genetic trend, (b) calendar year *t_k_* for non-genetic trend. *GRAIN_Y* Grain yield at 86 % dry matter, *PROTIN_C* Crude grain protein concentration [% of dry matter], *EXTRCT_C* Extract content in dry matter [%], *MALTNG_L* Malting loss, *FRIABLTY* Friability, *VISCOSTY* Viscosity, *PROTIN_S* Protein solution degree (Kolbach value), *ATTENUTN* Final attenuation degree

(b)


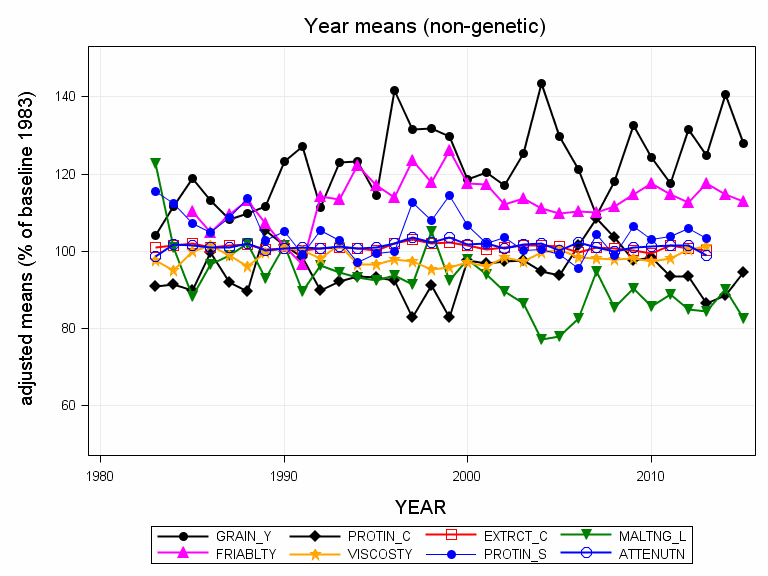

Supplement: Supplementary file 3 — Supplementary material 3 (DOCX 77 kb) [file 122_2017_2967_MOESM3_ESM.docx]
