# Supplementary material for: Breeding progress, genotypic and environmental variation and correlation of quality traits in malting barley in German official variety trials between 1983 and 2015
Source: Theor Appl Genet. 2017 Aug 18;130(11):2411–29. doi: 10.1007/s00122-017-2967-4 (PMC5641284; doi:10.1007/s00122-017-2967-4)
Supplement: Supplementary file 4 — Supplementary material 4 (DOCX 18 kb) [file 122_2017_2967_MOESM4_ESM.docx]

**Table S1**  Basic data

|  | Trait | Abbreviation | Unit of measurement | No. of  observations | Total no.  of varieties | No. of  years | No. of  locations | Percentage of  variety-year-location  combinations |
| --- | --- | --- | --- | --- | --- | --- | --- | --- |
| Yield components | Grain yield at 86 % dry matter | GRAIN_Y | dt ha^−1^ | 8594 | 187 | 33 | 73 | 1.66 |
|  | Single ear density | EAR_D | ears m^−2^ | 7881 | 186 | 33 | 71 | 1.56 |
|  | Number of kernels per ear | KERNLS_E | kernels ear^−1^ | 7190 | 186 | 33 | 68 | 1.49 |
|  | Thousand grain mass at 86 % dry matter | TGM | g (1000 kernels)^−1^ | 7816 | 187 | 33 | 71 | 1.54 |
| Grain quality | Grain fraction > 2.5 mm | GRAIN2.5 | % | 8026 | 187 | 33 | 73 | 1.55 |
|  | Hectoliter weight (test weight) | HECTOL_W | kg hl^−1^ | 7509 | 186 | 32 | 70 | 1.55 |
|  | Crude grain protein concentration [% of dry matter] | PROTIN_C | % | 8054 | 187 | 33 | 73 | 1.55 |
| Malting quality | Extract content in dry matter [%], | EXTRCT_C | % | 5857 | 156 | 31 | 69 | 1.54 |
|  | Malting loss | MALTNG_L | % | 6161 | 164 | 33 | 70 | 1.42 |
|  | Friability | FRIABLTY | % | 5266 | 151 | 31 | 56 | 1.74 |
|  | Viscosity | VISCOSTY | mPas | 5844 | 156 | 31 | 69 | 1.53 |
|  | Protein solution degree (Kolbach value) | PROTIN_S | % | 5867 | 156 | 31 | 69 | 1.54 |
|  | Final attenuation degree | ATTENUTN | % | 5862 | 156 | 31 | 69 | 1.54 |
