# Supplementary material for: Breeding progress, genotypic and environmental variation and correlation of quality traits in malting barley in German official variety trials between 1983 and 2015
Source: Theor Appl Genet. 2017 Aug 18;130(11):2411–29. doi: 10.1007/s00122-017-2967-4 (PMC5641284; doi:10.1007/s00122-017-2967-4)
Supplement: Supplementary file 5 — Supplementary material 5 (DOCX 24 kb) [file 122_2017_2967_MOESM5_ESM.docx]

**Table S2**: Spring barley varieties included in the study and the time period from which data were available

| **Reference**  **number** | **Variety**  **Name** | **Years of data** | |  | **Reference**  **number** | **Variety**  **Name** | **Years of data** | |
| --- | --- | --- | --- | --- | --- | --- | --- | --- |
|  |  | **from** | **to** |  |  |  | **from** | **to** |
| 504 | Aramir | 1983 | 1989 |  | 1354 | Ditta | 1989 | 1991 |
| 561 | Aura | 1983 | 1988 |  | 1364 | Meltan | 1989 | 1991 |
| 717 | Europa | 1983 | 1984 |  | 1390 | Alondra | 1990 | 1992 |
| 909 | Roland | 1984 | 1986 |  | 1413 | Katharina | 1990 | 1990 |
| 913 | Gunhild | 1983 | 1984 |  | 1414 | Marina | 1990 | 1990 |
| 967 | Arena | 1985 | 1991 |  | 1420 | Diamalta | 1991 | 1992 |
| 1018 | Golf | 1985 | 1990 |  | 1428 | Krona | 1992 | 1998 |
| 1051 | Hockey | 1985 | 1985 |  | 1442 | Chariot | 1991 | 1993 |
| 1095 | Cordula | 1983 | 1983 |  | 1496 | Polygena | 1991 | 1993 |
| 1098 | Ballerina | 1983 | 1985 |  | 1501 | Henni | 1992 | 1999 |
| 1100 | Amazone | 1983 | 1985 |  | 1523 | Thuringia | 1992 | 1994 |
| 1102 | Alexis | 1983 | 2004 |  | 1524 | Halla | 1992 | 1994 |
| 1113 | Lilo | 1983 | 1985 |  | 1543 | Scarlett | 1992 | 1999 |
| 1117 | Toga | 1983 | 1985 |  | 1546 | Brenda | 1992 | 1994 |
| 1121 | Regatta | 1984 | 1986 |  | 1565 | Sigrid | 1993 | 1995 |
| 1125 | Comtesse | 1984 | 1986 |  | 1576 | Orthega | 1993 | 1995 |
| 1128 | Cheri | 1984 | 1986 |  | 1582 | Barke | 1993 | 2006 |
| 1132 | Defra | 1984 | 1986 |  | 1598 | Bella | 1993 | 1995 |
| 1135 | Aphrodite | 1984 | 1986 |  | 1603 | Mentor | 1993 | 1995 |
| 1138 | Cirstin | 1984 | 1986 |  | 1612 | Madras | 1994 | 1996 |
| 1159 | Princesse | 1985 | 1987 |  | 1614 | Madonna | 1994 | 1996 |
| 1161 | Rumba | 1985 | 1987 |  | 1618 | Caminant | 1994 | 1996 |
| 1169 | Teo | 1985 | 1992 |  | 1641 | Escada | 1994 | 1996 |
| 1177 | Perun | 1985 | 1987 |  | 1643 | Hanka | 1994 | 1996 |
| 1179 | Phantom | 1985 | 1987 |  | 1668 | Baccara | 1995 | 1997 |
| 1205 | Stella | 1986 | 1988 |  | 1672 | Pasadena | 1995 | 2010 |
| 1208 | Baronesse | 1986 | 1995 |  | 1679 | Charlotte | 1995 | 1997 |
| 1213 | Ismene | 1986 | 1988 |  | 1680 | Ricarda | 1995 | 1997 |
| 1219 | Fink | 1986 | 1988 |  | 1702 | Extract | 1995 | 2001 |
| 1228 | Lenka | 1986 | 1988 |  | 1708 | Peggy | 1995 | 1997 |
| 1230 | Raisa | 1986 | 1988 |  | 1709 | Ria | 1995 | 1997 |
| 1234 | Steffi | 1986 | 1988 |  | 1710 | Madeira | 1995 | 1997 |
| 1249 | Nancy | 1987 | 1989 |  | 1725 | Aspen | 1996 | 1998 |
| 1251 | Nomad | 1987 | 1989 |  | 1726 | Chantal | 1996 | 1998 |
| 1253 | Maresi | 1987 | 1999 |  | 1739 | Sally | 1996 | 1998 |
| 1255 | Fergie | 1987 | 1989 |  | 1749 | Annabell | 1996 | 2004 |
| 1256 | Libelle | 1987 | 1989 |  | 1750 | Viskosa | 1996 | 1998 |
| 1265 | Claudine | 1987 | 1989 |  | 1781 | Eunova | 1997 | 1999 |
| 1266 | Cora | 1987 | 1989 |  | 1783 | Neruda | 1997 | 2002 |
| 1267 | Pompadour | 1987 | 1989 |  | 1786 | Havanna | 1997 | 1999 |
| 1269 | Meise | 1987 | 1989 |  | 1795 | Danuta | 1997 | 2002 |
| 1271 | Sissy | 1987 | 1989 |  | 1798 | Prolog | 1997 | 1999 |
| 1284 | City | 1988 | 1990 |  | 1823 | Saloon | 1997 | 1999 |
| 1304 | Olga | 1988 | 1990 |  | 1836 | Prestige | 1998 | 2000 |
| 1319 | Minna | 1988 | 1990 |  | 1854 | Birte | 1998 | 2000 |
| 1320 | Korinna | 1988 | 1990 |  | 1867 | Jacinta | 1998 | 2000 |
| 1338 | Otis | 1989 | 1991 |  | 1871 | Pewter | 1998 | 2000 |

continued

| **Reference**  **number** | **Variety**  **name** | **Years of data** | |  | **Reference**  **number** | **Variety**  **name** | **Years of data** | |
| --- | --- | --- | --- | --- | --- | --- | --- | --- |
|  |  | **from** | **to** |  |  |  | **from** | **to** |
| 1885 | Adonis | 1999 | 2001 |  | 2298 | Grace | 2006 | 2015 |
| 1897 | Ursa | 1999 | 2001 |  | 2323 | Steward | 2006 | 2009 |
| 1915 | Auriga | 1999 | 2007 |  | 2364 | KWS Aliciana | 2007 | 2009 |
| 1924 | Cellar | 1999 | 2001 |  | 2369 | KWS Bambina | 2007 | 2009 |
| 1926 | Braemar | 1999 | 2008 |  | 2385 | Despina | 2007 | 2009 |
| 1954 | Bellevue | 2000 | 2002 |  | 2395 | Propino | 2007 | 2014 |
| 1958 | Margret | 2000 | 2002 |  | 2398 | Sunshine | 2007 | 2011 |
| 1963 | Djamila | 2000 | 2002 |  | 2400 | Iron | 2007 | 2009 |
| 1978 | Denise | 2000 | 2002 |  | 2453 | Jazz | 2008 | 2010 |
| 1979 | Marnie | 2000 | 2003 |  | 2457 | SY Taberna | 2008 | 2010 |
| 1981 | Josefin | 2000 | 2002 |  | 2465 | Zeppelin | 2008 | 2010 |
| 1995 | Temperament | 2001 | 2003 |  | 2466 | Natasia | 2008 | 2010 |
| 1997 | Tocada | 2001 | 2003 |  | 2474 | Traveler | 2008 | 2010 |
| 2001 | Class | 2001 | 2003 |  | 2505 | Salome | 2009 | 2011 |
| 2003 | Berras | 2001 | 2003 |  | 2532 | Tesla | 2009 | 2012 |
| 2019 | Xanadu | 2001 | 2003 |  | 2537 | Catamaran | 2009 | 2011 |
| 2020 | Belana | 2001 | 2003 |  | 2540 | Passenger | 2009 | 2011 |
| 2021 | Simba | 2001 | 2009 |  | 2548 | Milford | 2009 | 2011 |
| 2039 | Carafe | 2001 | 2003 |  | 2563 | Kerstin | 2010 | 2012 |
| 2047 | Germina | 2002 | 2005 |  | 2567 | KWS Irina | 2010 | 2012 |
| 2052 | Mauritia | 2002 | 2004 |  | 2568 | KWS Thessa | 2010 | 2012 |
| 2070 | Isotta | 2002 | 2004 |  | 2573 | KWS Asta | 2010 | 2012 |
| 2076 | Beatrix | 2002 | 2004 |  | 2583 | Samitar | 2010 | 2012 |
| 2092 | Cristalia | 2002 | 2004 |  | 2585 | Melius | 2010 | 2012 |
| 2093 | Carvilla | 2002 | 2004 |  | 2587 | Vespa | 2010 | 2012 |
| 2094 | NFC Tipple | 2002 | 2004 |  | 2589 | Overture | 2010 | 2014 |
| 2110 | Westminster | 2003 | 2005 |  | 2595 | Britney | 2010 | 2012 |
| 2125 | Marthe | 2003 | 2015 |  | 2596 | Montoya | 2010 | 2013 |
| 2126 | Sophie | 2003 | 2005 |  | 2601 | Solist | 2010 | 2012 |
| 2136 | Power | 2003 | 2005 |  | 2606 | Avalon | 2010 | 2015 |
| 2137 | Sebastian | 2003 | 2005 |  | 2615 | KWS Dante | 2011 | 2013 |
| 2161 | Primadonna | 2004 | 2006 |  | 2627 | Gesine | 2011 | 2013 |
| 2164 | Lisanne | 2004 | 2006 |  | 2655 | Fortuna | 2011 | 2013 |
| 2174 | Ingmar | 2004 | 2006 |  | 2656 | Sydney | 2011 | 2013 |
| 2192 | Publican | 2004 | 2006 |  | 2663 | Endora | 2011 | 2013 |
| 2194 | Quench | 2004 | 2015 |  | 2678 | KWS Eileen | 2012 | 2014 |
| 2216 | Conchita | 2005 | 2008 |  | 2679 | KWS Grenada | 2012 | 2014 |
| 2221 | Victoriana | 2005 | 2007 |  | 2691 | Uta | 2012 | 2014 |
| 2224 | Henrike | 2005 | 2007 |  | 2694 | Paustian | 2012 | 2014 |
| 2226 | Jennifer | 2005 | 2007 |  | 2703 | RGT Planet | 2012 | 2015 |
| 2227 | Anakin | 2005 | 2007 |  | 2714 | Ventina | 2012 | 2014 |
| 2244 | JB Flavour | 2005 | 2007 |  | 2715 | Rheingold | 2012 | 2014 |
| 2257 | Streif | 2005 | 2009 |  | 2719 | Gladiator | 2012 | 2014 |
| 2258 | Kangoo | 2005 | 2007 |  | 2788 | Cervinia | 2013 | 2015 |
| 2282 | Yukata | 2006 | 2009 |  | 2794 | Crossway | 2013 | 2015 |
| 2291 | Concerto | 2006 | 2009 |  |  |  |  |  |
