# Supplementary material for: Breeding progress, genotypic and environmental variation and correlation of quality traits in malting barley in German official variety trials between 1983 and 2015
Source: Theor Appl Genet. 2017 Aug 18;130(11):2411–29. doi: 10.1007/s00122-017-2967-4 (PMC5641284; doi:10.1007/s00122-017-2967-4)
Supplement: Supplementary file 6 — Supplementary material 6 (DOCX 32 kb) [file 122_2017_2967_MOESM6_ESM.docx]

**Table S3** Change in sowing rate, date of sowing, date of harvest and annual average daily air temperatures during 1983/1991 and 2015 as estimated from regression analyses

| Variable | Unit | Data | | | Predicted values 1983 and 2015  based on linear trends | | | Estimate of linear trends | | |
| --- | --- | --- | --- | --- | --- | --- | --- | --- | --- | --- |
|  |  | Description | Source | Model | 1983 | 2015 | Difference | Slope | SE | P-value |
| Sowing rate | Kernels m^-2^ | 1316 year-location-combinations (sowing years: 1991-2015) | VCU trials | 1 | 347.4 | 319.6 | -27.8 | -0.869 | 0.101 | 0.000 |
| 01 Jan. to sowing date | Days | 1322 year-location-combinations (harvesting years: 1991-2015) |  |  | 87.7 | 83.2 | -4.5 | -0.141 | 0.257 | 0.583 |
| 01 Jan. to harvesting date | Days | 1273 year-location-combinations (harvesting years: 1991-2015) |  |  | 218.0 | 213.0 | -5.0 | -0.155 | 0.156 | 0.322 |
| Days between sowing and harvest | Days | 1273 year-location-combinations (harvesting years: 1991-2015) |  |  | 129.6 | 130.4 | 0.8 | 0.025 | 0.213 | 0.907 |
| Daily air temperature | °C | 45 annual average daily air temperatures in Germany (1971-2015) | DWD 2016 (Deutscher Wetterdienst) | 2 | 8.47 | 9.53 | 1.06 | 0.0331 | 0.0075 | <.0001 |

DWD (Deutscher Wetterdienst) (2016) German Meteorological Service

Climate Data Center (ftp://ftp-cdc.dwd.de/pub/CDC/regional_averages_DE/annual/air_temperature_mean/ regional_averages_tm_year.txt). Accessed 12 Oct 2016

Model 1:$y_{jk}=\varphi t_{k}+U_{k}+L_{j}+e_{jk}$, where $y_{jk}$is the observation in *j*th location and *k*th year, $\varphi$ is a fixed regression coefficient, *t_k_* is the continuous covariate for the calendar year, *U_k_* is a random deviation of *k*th year from linear trend, *L_j_* is the random effect of the *j*th location and is the residual error.

Model 2: $y_{k}=\mu+\omega t_{k}+e_{k}$ where *y_k_* is the annual average daily air temperature in year *k*, $\omega$ is a fixed regression coefficient, *t_k_* represents the calendar year, and $e_{k}$is the residual error.

*SE* standard error
