# Supplementary material for: Breeding progress, genotypic and environmental variation and correlation of quality traits in malting barley in German official variety trials between 1983 and 2015
Source: Theor Appl Genet. 2017 Aug 18;130(11):2411–29. doi: 10.1007/s00122-017-2967-4 (PMC5641284; doi:10.1007/s00122-017-2967-4)
Supplement: Supplementary file 7 — Supplementary material 7 (DOCX 23 kb) [file 122_2017_2967_MOESM7_ESM.docx]

**Genetic correlation coefficients derived from the extended model to take into account the reduction of malting time by one day in 2002**

We extended our model for estimating genetic and non-genetic trends ( Model (1) using Eq. 2 and 3) by a fixed model term “period” representing a time effect for the study period until 2001 and one for the period after reduction of malting time in 2002. We further allowed for an interaction term for period with genetic and non-genetic trends. From this model we calculated the genetic correlation coefficients as described in Materials and methods. The results in Table S4 confirmed genetic correlations as shown in Table 3 for grain yield between malting traits, and also among malting traits.

**Table S4** Correlations

|  | GRAIN_Y | | EXTRCT_C | | MALTNG_L | | FRIABLTY | | | VISCOSTY | | PROTIN_S | | | ATTENUTN |
| --- | --- | --- | --- | --- | --- | --- | --- | --- | --- | --- | --- | --- | --- | --- | --- |
| N | 187 | | 156 | | 164 | | 151 | | | 156 | | 156 | | | 156 |
| Traits | Genetic correlation coefficients ρ_g_ for extended model | | | | | | | | | | | | | | |
| GRAIN_Y | 1 | |  | |  | |  | | |  | |  | | |  |
| EXTRCT_C | -0.56 |  | 1 | |  | |  | | |  | | |  | |  |
| MALTNG_L MALTNG_L | -0.34 |  | 0.51 |  | 1 | | |  | |  | | |  | |  |
| FRIABLTY | -0.35 |  | 0.38 |  | 0.42 |  | | 1 | |  | | |  | |  |
| VISCOSTY | 0.30 |  | -0.58 |  | -0.50 |  | | -0.82 |  | 1 | | |  | |  |
| PROTIN_S | -0.42 |  | 0.55 |  | 0.51 |  | | 0.57 |  | -0.58 |  | | 1 | |  |
| ATTENUTN | -0.23 |  | 0.36 |  | 0.32 |  | | 0.47 |  | -0.58 |  | | 0.26 |  | 1 |

*N:* Number of varieties .

^ns^ not significant different from zero at 1% level.

*GRAIN_Y* Grain yield at 86 % dry matter, *EXTRCT_C* Extract content in dry matter [%], *MALTNG_L* Malting loss, *FRIABLTY* Friability, *VISCOSTY* Viscosity, *PROTIN_S* Protein solution degree (Kolbach value), *ATTENUTN* Final attenuation degree
